# Disulfide‐Bridged Ru Complex–Mediated Photo‐Disulfidptosis for Colorectal Cancer Therapy

**DOI:** 10.1002/advs.202516046

**Published:** 2026-02-16

**Authors:** Simeng He, Wendong Jin, Jiaojiao Pang, Xiaoting Gong, Shixian Cao, Duo Mao, Yuguo Chen, Kang‐Nan Wang, Bin Liu

**Affiliations:** ^1^ Department of Emergency Medicine Qilu Hospital of Shandong University Jinan China; ^2^ State Key Laboratory of Crystal Materials Shandong University Jinan China; ^3^ Department of Chemical and Biomolecular Engineering National University of Singapore Singapore Singapore; ^4^ Institute of Precision Medicine The First Affiliated Hospital of Sun Yat‐Sen University Sun Yat‐Sen University Guangzhou China

**Keywords:** collaborative treatment, colon cancer treatment, disulfidptosis, photoactivated

## Abstract

Despite numerous therapeutic strategies targeting specific programmed cell death pathways, effectively eliminating malignant colorectal cancer (CRC) remains a significant challenge. Disulfidptosis, a newly identified form of cell death, is characterized by rapid NADPH depletion and abnormal disulfide bond formation in cytoskeletal proteins in cells with high SLC7A11 expression under glucose starvation. Given the aberrant expression of SLC7A11 and the hypermetabolic phenotype of CRC cells, targeting the disulfidptosis pathway offers a promising therapeutic approach for CRC treatment. Here, we developed a binuclear ruthenium complex, RuSSRu, bridged by a disulfide bond, to effectively induce the disulfidptosis pathway in CRC cells. Under two‐photon excitation, RuSSRu generates reactive oxygen species (ROS), leading to lysosomal damage and initiating cellular escape mechanisms. Crucially, this process triggers a cascade of metabolic disruptions, including an accumulation of disulfide bonds and ROS‐induced NADPH depletion, ultimately resulting in cytoskeletal collapse and disulfidptosis. The synergistic interaction between disulfidptosis and lysosomal damage‐induced apoptosis amplified tumor cell death, unveiling a novel mechanism and a versatile therapeutic strategy platform for CRC management.

## Graphical abstract

In this work, a binuclear ruthenium complex, RuSSRu was developed for efficient colorectal cancer (CRC) treatment. The synergistic interaction between disulfidptosis and lysosomal damage‐induced apoptosis induced by RuSSRu amplified tumor cell death. This innovative approach offers a potent therapeutic strategy for CRC.

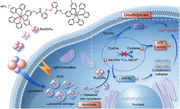

## 1 Introduction

Colorectal cancer (CRC) is the third most prevalent cancer worldwide, marked by high incidence and mortality rates, with approximately 0.9 million deaths globally each year [1]. Currently, surgery, radiotherapy, and chemotherapy are the primary clinical treatments; however, for CRC, these approaches have shown limited efficacy in preventing recurrence and metastasis, and the 5‐year survival rate has seen minimal improvement [2]. Drug development efforts now focus on activating tumor‐specific programmed cell death pathways, such as apoptosis, pyroptosis, and ferroptosis, to induce tumor necrosis [3, 4]. However, due to tumor resistance and the influence of the tumor microenvironment, current treatments often fall short in effectively suppressing tumor recurrence and further metastasis [5, 6]. Therefore, exploring novel mechanisms of tumor cell death and designing new therapeutic strategies has become a priority in CRC research.

**FIGURE 1:**
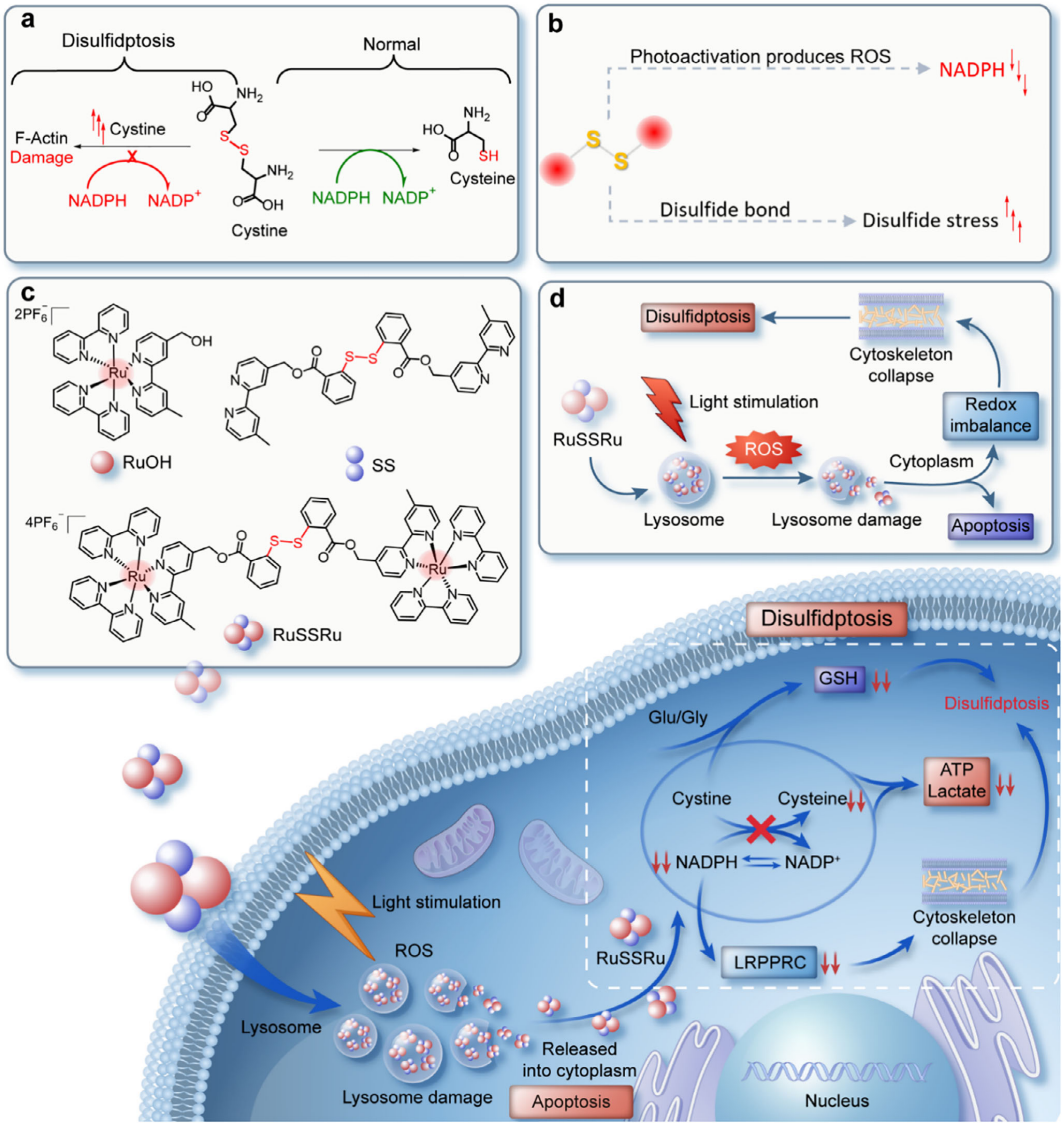
Scheme illustrating the preparation and function of RuSSRu. (a) The metabolic processes involved in cystine in normal and disulfidptosis states, NADPH exhaustion, and abnormal accumulation of disulfides are key factors inducing disulfidptosis. (b) Disulfide bond‐containing photosensitizers synergize NADPH oxidation and induce disulfide stress. (c) Schematic diagram of the chemical structures of RuSSRu and its control molecules. (d) The synergistic therapeutic effect of RuSSRu combining PDT, apoptosis, and disulfidptosis.

Disulfidptosis represents a newly discovered form of cell death that is distinct from established programmed cell death pathways [7, 8, 9, 10]. This process is defined by abnormal cystine accumulation, driven by high expression of solute carrier family 7 member 11 (SLC7A11) under glucose‐starvation conditions, which promotes abnormal disulfide bond formation within actin cytoskeletal proteins, ultimately resulting in actin‐network collapse and cell death [11, 12]. Under normal conditions, the reduced form of nicotinamide adenine dinucleotide phosphate (NADPH) provides critical reducing capacity to offset disulfide toxicity and maintain cell survival [13, 14]. In cancer cells with aberrant SLC7A11 expression and hypermetabolic activity, such as colon cancer cells, the high uptake of cystine and the rate of cystinyl reduction to cysteine consume large amounts of NADPH [15, 16]. Once NADPH exhaustion or abnormal accumulation of disulfides could induce disulfidptosis [9, 17]. Although no therapeutic agents have yet been developed to specifically target disulfidptosis in cancer treatment, this mechanism offers a promising foundation for designing novel materials and strategies that leverage disulfidptosis for colon cancer therapy (Figure 1a).

The ROS produced has strong oxidative activity, which is bound to undergo redox reactions with reducing NADPH, leading to its depletion and inactivation [18]. Under light‐activated conditions, photosensitive molecules can efficiently generate ROS [18, 19], accelerating the depletion of NADPH and inducing oxidative damage in tumor cells. In addition, introducing disulfide bond units into anti‐tumor molecules can further enhance the abnormal accumulation of disulfide bonds in cancer cells. These exogenous and abnormally accumulated disulfide bonds lead to protein misfolding and functional abnormalities, thereby activating the process of disulfidptosis [17]. Thus, by leveraging the characteristics of glucose starvation in CRC cells, photosensitive molecules with disulfide bonds can ablate tumor cells via photodamage, while inducing disulfidptosis. This dual action (Figure 1b) may significantly enhance the therapeutic efficacy against CRC.

Ruthenium complexes are widely recognized for their use as photocatalysts and photosensitizers, owing to their exceptional photocatalytic properties and ROS generation capabilities [20]. Notably, several ruthenium‐based complexes, including KP1019, its more soluble derivative KP1339, BOLD‐100, NAMI‐A, and TLD‐1433, have recently entered clinical trials, driven by their excellent biocompatibility and antitumor efficacy [21, 22, 23, 24]. Inspired by these advancements, we employed RuOH, a ruthenium complex containing a bipyridine ligand functionalized with a hydroxymethyl group, as a structural control [25], and carried out further engineering design: first, the hydroxymethyl of bipyridine was equipped with a disulfide bond unit via an esterification reaction to obtain the ligand SS. Then, the ligand SS was used as a bridge to assemble two ruthenium complexes together to obtain a binuclear ruthenium complex containing a disulfide bond, RuSSRu (Figure 1c). The ROS produced by RuSSRu under light stimulation increases the permeability of the lysosomal membrane and damages the lysosomes, further inducing cell apoptosis. The disulfide unit equipped in RuSSRu also further exacerbates the irreversible accumulation of disulfides in cells, which leads to disulfide stress and downregulates LRPPRC (leucine‐rich pentatricopeptide repeat‐containing protein), playing an important role in regulating the interaction of the cytoskeleton with various cellular processes, ultimately contributing to the expression patterns that trigger disulfidptosis [26]. (Figure 1d). This process, in synergy with light induced lysosomal damage (apoptotic pathway), significantly improves the anti‐tumor efficacy of RuSSRu. Considering the characteristics of the superficial tumor model of CRC, an in situ injection scheme was adopted in this work [27]. As the compound can only be activated with blue light, and it is difficult to effectively penetrate the tumor tissue to produce ROS under conventional single‐photon excitation, two‐photon irradiation was used to achieve satisfactory anti‐tumor effects. This work integrates the photoactivated‐induced apoptosis with disulfide stress‐induced disulfidptosis, greatly improving the killing effect of hypoxic tumors.

## 2 Results and Discussion

### 2.1 Photophysical Properties Characteristics of RuSSRu

**FIGURE 2:**
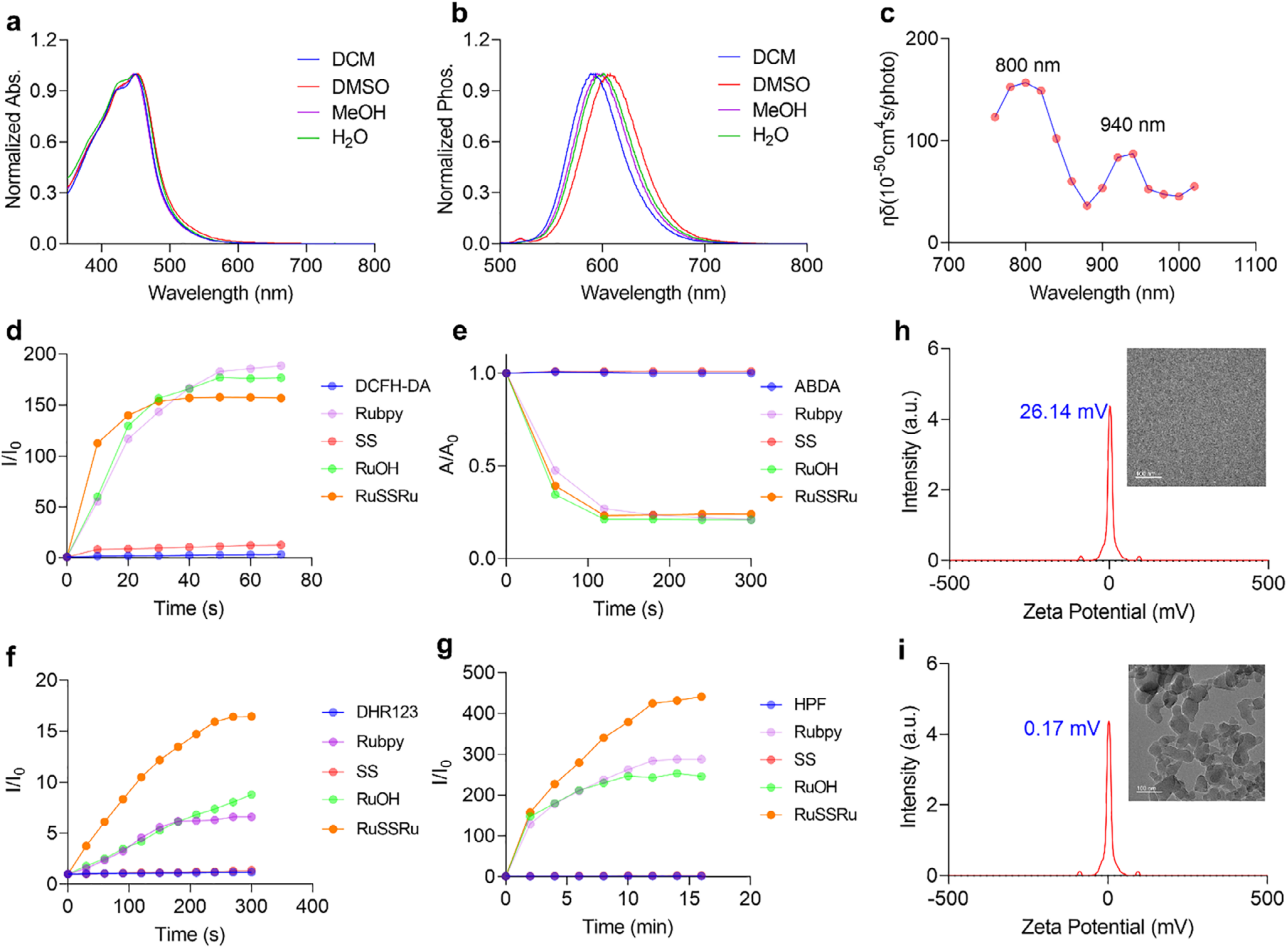
Evaluation of photophysical and photosensitivity properties of RuSSRu. (a,b) Normalized UV/Vis spectra (a) and phosphorescence emission spectra (b) of RuSSRu (10 µm) measured in CH_2_Cl_2_, DMSO, H_2_O, and MeOH, respectively, at 25°C, λ_ex_ = 425 nm. (c) Two‐photon absorption cross section of RuSSRu in H_2_O. (d) Comparison of the relative fluorescence intensity of DCFH‐DA alone and DCFH‐DA with different control molecules or RuSSRu for overall ROS detection. e) Comparison of the decomposition of ABDA alone and ABDA with different control molecules or RuSSRu for ^1^O_2_ detection. f) Comparison of the relative fluorescence intensity of DHR123 alone and DHR123 with different control molecules or RuSSRu for O_2_^−•^ detection. g) Comparison of the decomposition of HPF alone and HPF with different control molecules or RuSSRu for OH^−•^ detection. In the ROS experiments, white light with a wavelength range of 350–750 nm was used as the illumination source, and the light intensity was set for 30 mW/cm^2^. Control molecules: RuOH, SS, commercial photosensitizers [Ru(bpy)_3_]Cl_2_ (abbreviated as Rubpy). (h,i) The Z‐potential and TEM images of RuSSRu at a concentration of 10 µm (h) and 1 mM (i), respectively.

The synthesis of the ligand SS and the RuSSRu, as well as their characterization spectra (HRMS, ^1^H NMR, ^1^ ^3^C NMR, and HPLC), are presented in Scheme S1 and Figures S1–S7 in the Supporting Information. RuSSRu exhibits a broad absorption band at 400–600 nm, with the molar absorptivity in different solvents ranging 0.24–0.28 *10^5^ M^−1^ cm^−1^, and an emission peak around 600 nm (Figure 2a,b). The photophysical properties of RuSSRu are summarized in Table S1. As shown in Figure 2c, although the two‐photon absorption cross‐sections of RuSSRu increase significantly around both 800 nm and 940 nm, the cross‐section near 800 nm is larger, reaching 156.86 GM. The >900 nm region corresponds to a strong overtone/combination absorption band of water (O─H stretching vibrations). This enhanced water absorption attenuates the excitation beam during propagation, reducing the effective photon flux reaching the focal volume. Additionally, water‐induced thermal effects may further deteriorate the focal quality under femtosecond excitation. Therefore, in subsequent experiments, an 808 nm laser—closest to this wavelength—was selected as the excitation source. As anticipated, RuSSRu demonstrates outstanding two‐photon absorption properties (Figure 2c), supporting its potential anti‐tumor effects via two‐photon photoactivation.

A detailed evaluation of both RuOH and RuSSRu indicates that they exhibit efficient generation of type I and type II ROS [28]; for RuOH, its ROS generation capability is comparable to that of [Ru(bpy)_3_]Cl_2_. In contrast, the ligand SS shows negligible ROS production (Figure 2d–g). Considering the 3D structural characteristics of the Ru(II) complexes [29], we evaluated the particle size of RuSSRu at both the working concentration (10 µm) and a higher concentration (1 mM), with corresponding zeta potentials of 26.14 and 0.17 mV, respectively. TEM analysis further confirmed these observations, revealing no aggregation at the working concentration and a size distribution of approximately 100 nm at the higher concentration (Figure 2h,i). The result of DLS confirmed the size distribution of RuSSRu in an aggregation (Figure S8). Furthermore, the stability of RuSSRu in biological environments was examined by HPLC. The complex retained over 95% of its original chromatographic peak area after incubation in cell culture medium (DMEM) at 37°C, indicating excellent stability and the absence of significant decomposition or ligand dissociation (Figure 2c). These results suggest that RuSSRu remains molecularly stable and well‐dispersed under physiological conditions suitable for cell experiments.

**FIGURE 3:**
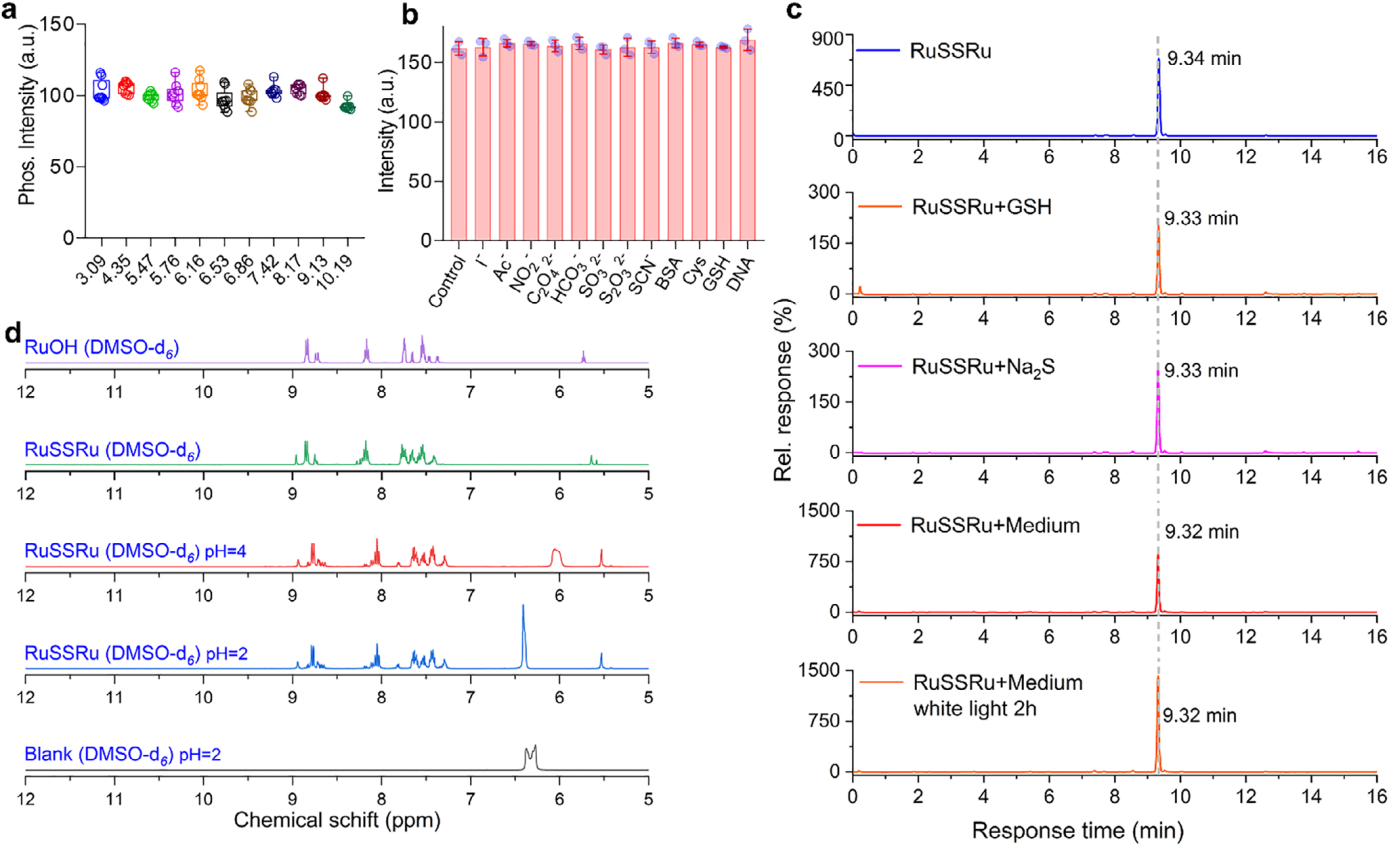
Stability evaluation of RuSSRu. (a) The phosphorescence intensity of the RuSSRu in PBS with different pH. (b) Phosphorescence intensity changes of RuSSRu in the presence of various biologically relevant species. (c) Representative HPLC spectra of RuSSRu, RuSSRu+GSH, RuSSRu+Na_2_S, and RuSSRu with cell culture medium under dark or white light (400–700 nm) with an intensity of 100 mW/cm^2^ for 2 h conditions recorded under identical chromatographic conditions (mobile phase: acetonitrile/water with 0.1% TFA, detection at 254 nm). (d) ^1^H NMR spectra of RuSSRu and control molecule (RuOH) in buffer solution with different pH, the molecules were subjected to NMR analysis after being stabilized in the different solvent environments for 12 h. The concentrations of the probes, as well as the experimental protocol, are detailed in the Materials and Methods Section.

As shown in Figure 3a, RuSSRu has stable phosphorescence in the range of pH = 3.09 to 10.19. RuSSRu exhibits negligible changes in fluorescence intensity in the presence of various biologically active species, indicating its excellent photostability and reliability in complex biological environments (Figure 3b). As shown in Figure 3c and Figure S9, HPLC‐based stability analyses were also conducted to evaluate the integrity of RuSSRu under various conditions, including in the presence of GSH, in cell culture medium (under both dark and light irradiation). The results demonstrate that RuSSRu remains chemically stable across a range of physiological and external conditions, supporting its suitability for biological applications. In addition, the chemical stability of RuSSRu was further evaluated using ^1^H NMR after stabilization in buffer solutions of varying pH for 12 h (Figure 3d). Upon comparison, RuSSRu has no obvious chemical shift or new peak generation in the acidic solution of pH = 2 and 4, indicating that it is stable in an acidic environment, such as the tumor microenvironment. These results collectively demonstrate that RuSSRu exhibits excellent stability in both solution and cell culture environments, without undergoing degradation during cellular uptake.

### 2.2 Lysosomal Localization and Light‐Induced Increase in Lysosomal Membrane Permeability

**FIGURE 4:**
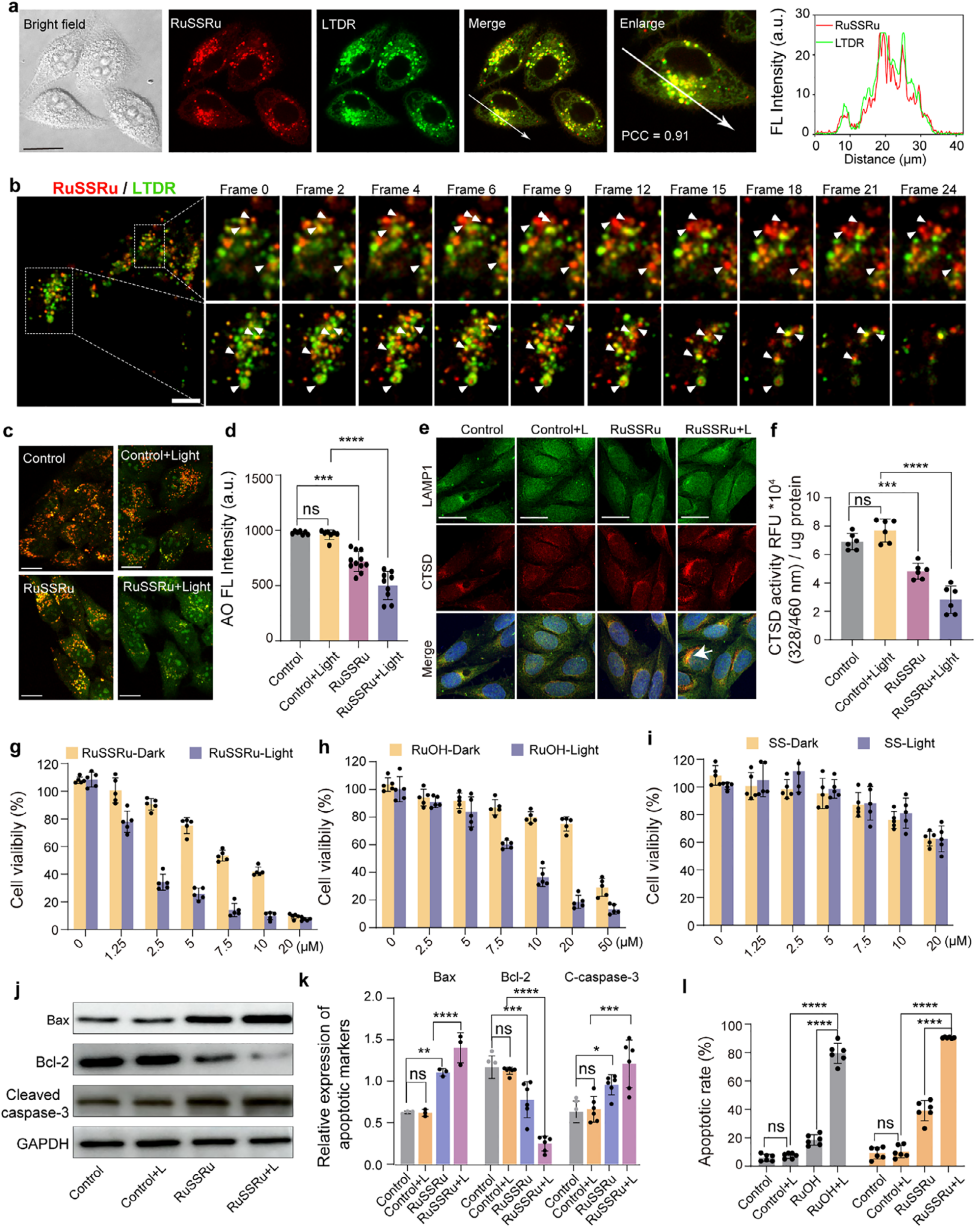
Light‐induced increase in lysosomal membrane permeability of RuSSRu and cytotoxicity evaluation of RuSSRu in vitro. (a) Co‐localization between RuSSRu (2 µm) and lysosome probe LTDR (200 nM), λ_ex/em_ = 405 nm / 600 ± 20 nm for RuSSRu (red); λ_ex/em_ = 633 nm / 650 ± 10 nm for LTDR (pseudo‐green). PCC: Pearson correlation coefficient. Scale bar = 20 µm. (b) The dynamic process tracking of RuSSRu escapes from lysosomes under light exposure using a laser scanning confocal microscope. Scale bar = 5 µm. (c,d) Representative images of acridine orange (AO) staining (c) and its fluorescence quantification of the red channel (d). For AO staining, green channel: λex/em = 488 nm / 530 ± 20 nm, red channel: λex/em = 561 nm / 640 ± 20 nm. Scale bar = 20 µm. (e) Immunofluorescence co‐localization of CTSD and LAMP1. Scale bar = 20 µm. (f) Quantification statistics of the enzyme activity of CTSD. (g–i) Cell viability of MC38 cells incubated with RuSSRu (g), RuOH (h), and SS (i) for 24 h with/without light irradiation. j‐k) Western blotting and quantification of apoptosis‐associated markers (Bax, *n* = 3, Bcl‐2, *n* = 6, cleaved caspase‐3, *n* = 6) after being treated with RuSSRu, under dark or light irradiation stimulation. (l) Cells' apoptotic rate after treated with RuOH and RuSSRu, under dark or light irradiation conditions for 24 h. Error bar indicates Standard Deviation (SD). ^*^ *P* < 0.05, ^**^ *P* < 0.01, ^***^ *P* < 0.001, ^****^ *P* < 0.0001.

Considering the structure of the RuSSRu, we evaluated its organelle‐targeting properties in cells using the commercial lysosomal targeting probe Lyso‐Tracker^TM^ Deep Red (LTDR) and mitochondria targeting probe Mito‐Tracker^TM^ Deep Red (MTDR) for co·localization analysis. As anticipated, the red phosphorescence from RuSSRu perfectly overlapped with the fluorescence of LTDR, yielding a Pearson colocalization coefficient (PCC) of 0.91. In contrast, the PCC with MTDR was only 0.14, indicating that RuSSRu is predominantly enriched in lysosomes (Figure 4a; Figure S10). We also used stimulated emission depletion (STED) microscopy to track the dynamic process of RuSSRu in lysosomes after light illumination. As shown in Figure 4b, with light stimulation, the overlay (yellow region) of red RuSSRu and green (pseudo‐color) LTDR gradually disappeared, while the red and green fluorescence from RuSSRu and LTDR, respectively, began to emerge separately (Figure 4b). This indicates that RuSSRu could progressively escape from the lysosome.

Given the lysosomal targeting properties of RuSSRu and its ability to escape from lysosomes under light exposure, we conducted a comprehensive evaluation of the impact of RuSSRu on lysosomal function. The lysosomal membrane permeability (LMP) was initially assessed using acridine orange (AO) staining and was quantified by a microplate reader. AO can enter lysosomes in a pH‐dependent manner, and present red fluorescence in intact lysosomes; when LMP increases, AO is released into the cytoplasm with bright green fluorescence [30]. Compared to the control and dark conditions, green fluorescence intensity was increased in the RuSSRu‐treated group, indicating LMP was significantly elevated (Figure 4c,d). Simultaneously, the leakage of lysosomal hydrolases outside the membrane was assessed to indirectly evaluate lysosomal membrane integrity. The release of cathepsin D (CTSD) [31] from the lysosomal lumen into the cytoplasm was increased when RuSSRu was exposed to laser stimuli, which was consistent with the results of AO staining (Figure 4e). Notably, during this process, RuSSRu reduced CTSD activity by approximately 30% in the dark and nearly 60% under light stimulation (Figure 4f). Furthermore, we evaluated lysosomal biogenesis by examining the nuclear translocation of TFEB, a master transcriptional factor responsible for regulating lysosomal biogenesis [32, 33]. The nuclear translocation of TFEB did not change significantly between groups and the mRNA levels of genes associated with lysosomal biogenesis (*Ctsb*, *Ctsd*, *Lamp1*, and *Clcn7*) were also not significantly altered, indicating no significant effects upon lysosomal biogenesis (Figure S11). These results suggested that RuSSRu might compromise the lysosome function and increase the lysosome membrane permeability, but has no impact on lysosomal biogenesis.

### 2.3 In Vitro Cytotoxicity Assessment of RuSSRu

To further evaluate the therapeutic potential of RuSSRu in tumors, its biocompatibility and cytotoxicity in vitro were first evaluated. The viability of MC38 cells incubated with different concentration gradients of RuSSRu, and two intermediates RuOH and SS, along with the control compound [Ru(bpy)_3_]Cl_2_ were determined by the CCK8 assay. In detail, after incubating with the compound for 6 h, cells were or were not exposed to light irradiation at 425 nm for 10 min (30 mW cm^−2^) and cultured for another 12 or 24 h. Under light irradiation, RuSSRu presented a dose‐dependent antitumor effect, with an IC50 value of 2.19 µm for 24 h, which is significantly higher than that in dark conditions (IC50 = 8.18 µm). In addition, its antitumor activity was significantly higher than that of the control molecules RuOH, [Ru(bpy)_3_]Cl_2_, and SS, both under light and dark conditions (Figure 4g–i; Figures S12 and S13). It is important to note that the intracellular ruthenium content was maintained at almost the same level when cells were incubated with RuOH or RuSSRu for 12 h (Figure S14). To evaluate the universality of RuSSRu in killing colorectal cancer cells, we further investigated its effect on the human colon cancer‐derived cell line HCT116 and similar results were also observed (Figure S15). Furthermore, the scratch assay results indicated that the migration rate in the RuSSRu with light treatment group was slower than that in the control group (Figure S16), further demonstrating the efficiency of the disulfide‐bridged Ru complex for CRC treatment via photoactivation. Both ruthenium complexes can photoactivate the fluorescence of DCFH‐DA, indicating a significant increase in intracellular ROS after light irradiation (Figure S17).

Malondialdehyde (MDA) is a biomarker of lipid peroxidation, reflecting the levels of intracellular oxidative stress [34]. The increase in MDA expression in the RuSSRu‐light‐treated group indicated that cells co‐incubated with RuSSRu and stimulated with light irradiation had higher oxidative stress levels compared with the control group (Figure S18). Cell colony formation assays demonstrated that light‐activated RuSSRu significantly suppressed colony formation, confirming its long‐term antiproliferative effect (Figure S19). Western blot analysis of apoptosis‐related proteins revealed that RuSSRu effectively induced apoptosis [35]. Specifically, compared to the control and dark conditions, RuSSRu under light irradiation markedly upregulated the expression of pro‐apoptotic proteins Bax and cleaved caspase‐3, while downregulating the anti‐apoptotic protein Bcl‐2 (Figure 4j,k). Furthermore, apoptosis induced by RuOH and RuSSRu was evaluated using Annexin V–PI staining. Combined flow cytometry analysis demonstrated that both RuOH and RuSSRu triggered similar levels of apoptosis under light irradiation, with apoptotic ratios exceeding 90% (Figure 4l; Figure S20). Notably, despite these comparable apoptotic rates, RuSSRu exhibited significantly greater efficacy in tumor cell ablation than RuOH under equivalent Ru cellular uptake conditions (Figure S14), suggesting that additional mechanisms beyond apoptosis may contribute to its enhanced therapeutic effect.

### 2.4 RuSSRu Trigger Disulfidptosis Following Lysosomal Release

To explore the mechanisms behind the anti‐tumorigenic effects of RuSSRu, we focused on the role of disulfide bonds. Disulfidptosis is a newly identified form of cell death induced by excessive formation of intracellular disulfides, which is characterized by F‐actin cytoskeleton collapse and followed by cell membrane rupture, accompanied by alterations of metabolism [7]. The increased lysosomal membrane permeability caused by ROS allows RuSSRu to freely traverse the lysosome. Once it is released into the cytoplasm, it may trigger disulfidptosis mediated by excessive disulfide bonds. We thus evaluated disulfidptosis‐related biomarkers (such as NADPH levels, cysteine levels, ATP contents, lactate levels, and GSH concentrations) [7, 8, 17] to investigate whether the antitumor effect of RuSSRu involves disulfidptosis.

**FIGURE 5:**
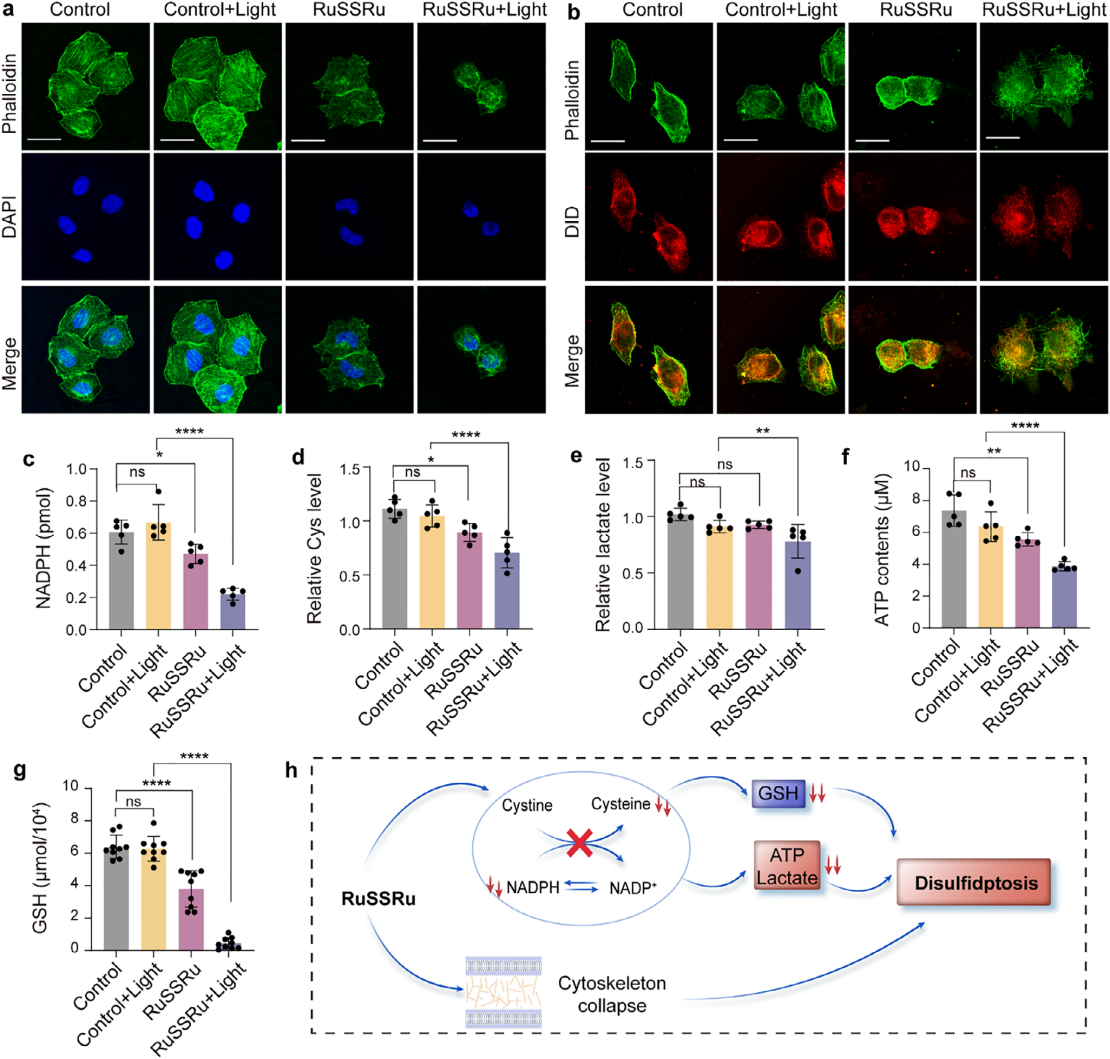
Changes of cytoskeleton and intracellular metabolism during disulfidptosis. (a) Fluorescent staining of F‐actin using Alexa 488 conjugated phalloidin (λ_ex/em_ = 488 nm /518 ± 20 nm) in MC38 cells cultured with/without RuSSRu, under dark or light irradiation conditions. Scale bar = 20 µm. The DAPI was used to label cell nuclei (λ_ex/em_ = 405 nm /488 ± 20 nm). (b) Fluorescent staining of F‐actin using Alexa 488 conjugated phalloidin (λ_ex/em_ = 488 nm /518 ± 20 nm) and staining of cell membrane using DID (λe_x/em_ = 633 /650±10 nm) of MC38 cells under different treatments. Scale bar = 20 µm. (c–g) NADPH levels (c), Cellular cysteine (Cys) levels (d), lactate levels (e), ATP contents (f) and GSH concentrations (g) under different groups. (h) Schematic of the disulfidptosis induced by RuSSRu. Error bar indicates Standard Deviation (SD). ^*^ *P* < 0.05, ^**^ *P* < 0.01, ^***^ *P* < 0.001, ^****^ *P* < 0.0001.

Phalloidin modified with fluorescent dyes is widely used as an F‐actin staining agent [36]. Here, we employed this technique to assess the integrity of the F‐actin cytoskeleton in cells incubated with RuSSRu under different conditions. Compared with controls and dark conditions, the cells incubated with RuSSRu under light irradiation showed significant cytoskeletal disruption and collapse (Figure 5a). Co‐staining of phalloidin and DID‐labeled cell membrane further showed that F‐actin cytoskeletal derangement and crumpled or ruptured plasma membrane occurred during the process (Figure 5b). These morphological alterations were not observed in RuOH‐stimulated cells (Figure S21).

Dysregulated energy metabolism has been identified as another critical characteristic of disulfidptosis [7]. The imbalance in the conversion between cystine‐cysteine and NADPH‐NADP^+^ is a distinctive feature of disulfidptosis. Since the ROS produced by RuSSRu may undergo redox reaction with reducing NADPH, leading to its depletion and inactivation, we evaluated the cellular NADPH and cysteine levels among groups. As expected, compared with the effect of RuSSRu under dark conditions, the free NADPH and cysteine levels were significantly decreased in the RuSSRu with light‐treated group (Figure 5c,d). Moreover, the glucose metabolic pathway was evaluated. The lactate levels reflecting glycolysis were significantly decreased in RuSSRu with the light‐treated group relative to those of the controls (Figure 5e), indicating excess disulfide bonds could hinder the activities of key enzymes in the glycolysis metabolic pathway. Conversely, ATP production was reduced when cells were stimulated by RuSSRu combined with light irradiation (Figure 5f), which can be attributed to impaired glycose metabolism. Furthermore, GSH contents, reflecting the intracellular antioxidant capacity, were determined, and the results showed that GSH contents were significantly decreased in the RuSSRu with light‐treated group relative to controls (Figure 5g). These alterations complied with the metabolic characteristics of disulfidptosis (Figure 5h). Together with the morphological changes, we preliminarily concluded that RuSSRu‐induced disulfidptosis promotes cell death.

### 2.5 Therapeutic Efficacy of RuSSRu in Colon Cancer

Inspired by the satisfactory antitumor efficacy in vitro, we investigated the treatment efficiency of RuSSRu in vivo. The tumor‐bearing mice model was constructed by subcutaneously inoculating 1 × 10^6^ MC38 cells into the loose connective tissue of the left axilla. To evaluate the antitumor activity of RuSSRu objectively, the xenograft nude mouse was randomly divided into five groups, including Control‐Dark, Control‐Light, Cisplatin, RuSSRu‐Dark, and RuSSRu‐Light groups (n = 6/group), and recorded the tumor volume/mass for different groups in a 28‐day therapeutic period.

**FIGURE 6:**
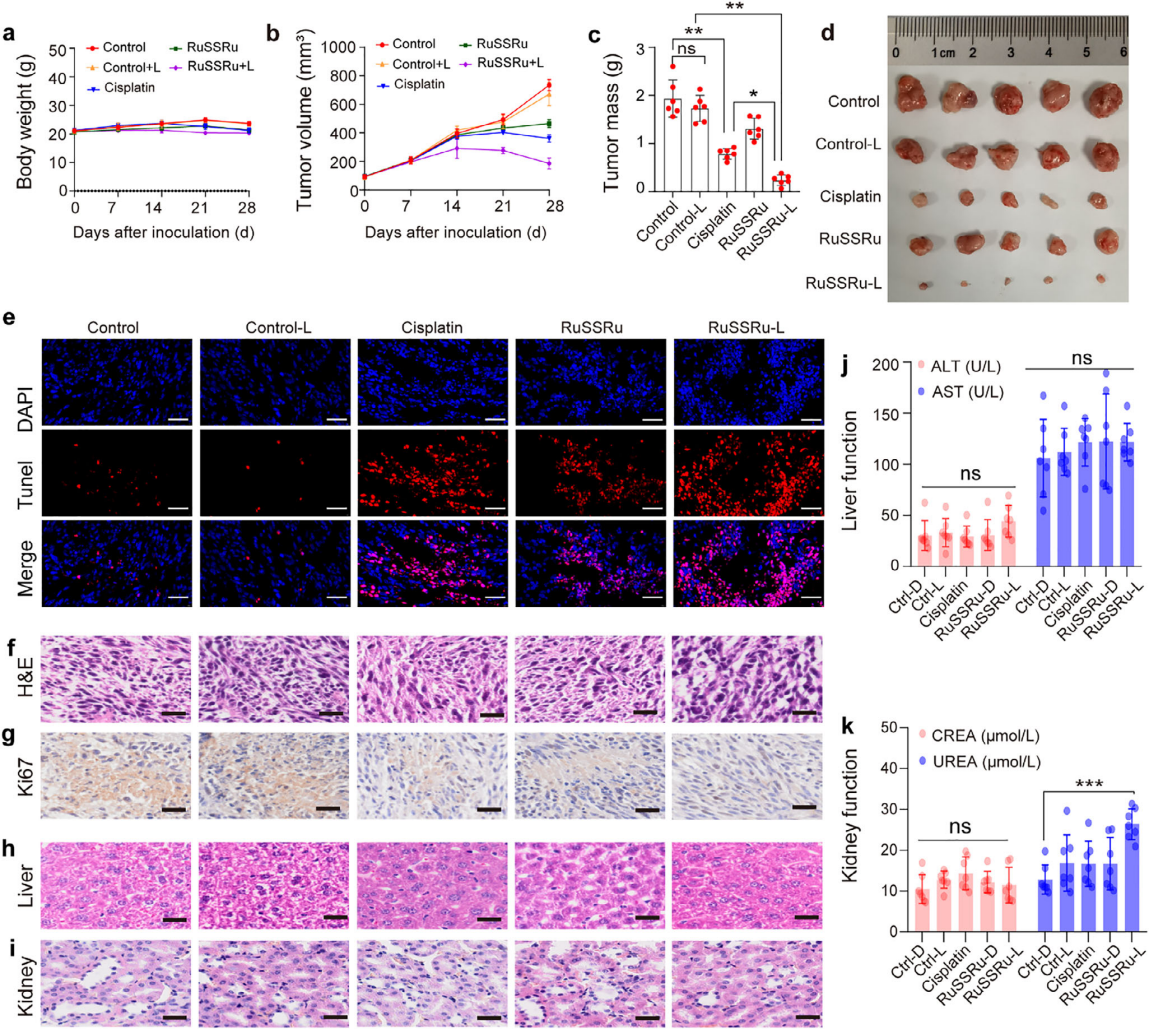
Therapeutic effect of RuSSRu in colon tumor in vivo. (a–c) The curve of body weight (a), tumor volume (b), and tumor weight (c) of mice during the therapeutic period. (d) Tumors isolated from mice after treatments. (e–g) TUNEL staining (e), H&E staining (f), and Ki67 immunohistochemistry analysis (g) of tumor sections after treatments. Scale bar = 40 µm. (h,i) Representative images of H&E staining of liver (h) and kidney (i). Scale bar = 40 µm. (j,k) In vivo biological safety was assessed by serum biochemical analysis after the treatment course, including liver (j), and kidney function (k). Error bar indicates Standard Deviation (SD). ^*^ *P* < 0.05, ^**^ *P* < 0.01, ^***^ *P* < 0.001.

Considering the structural and dimensional characteristics of RuSSRu, the biodistributions of RuSSRu in main organs (heart, lung, liver, spleen, and kidney) and tumor sites were first assessed. RuSSRu were administrated via the tail vein for 6h, the mice were euthanized, then we excised the main organs and tumor tissues and observed using an in vivo imaging system (IVIS). The phosphorescence signal of the complex was detectable predominantly in tumors, suggesting that RuSSRu is highly specific in tumor tissues rather than other major organs (Figure S22). Due to the characteristics of the superficial tumor model of CRC, an in situ tumor injection scheme was adopted in this work. During the 28‐day treatment, the body weight of mice in each group was recorded and not significantly altered (Figure 6a). Macroscopically, the tumor growth in 5 groups was almost unaltered within the first 14 days, but was suppressed after the second 14 days of treatment (Figure 6b,c). As two‐photon irradiation can significantly enhance tissue penetration [37], upon two‐photon irradiation (808 nm, 40 mW cm^−2^) for 10 min, the tumor growth in the RuSSRu‐Light group was markedly inhibited relative to that of the control group (blank) and cisplatin/RuSSRu‐Dark group (Figure 6d; Figure S23). These results were also confirmed by fluorescence imaging in vivo, statistical data on tumor volume/mass, and ex vivo tumor images (Figure 6b–d). Microscopically, we examined the apoptotic tumor cells in the tumor tissue by TUNEL staining. For the treatment groups, compared with cisplatin/RuSSRu‐dark group, the tumor cells from the RuSSRu+Light group had the greatest amount of apoptosis (Figure 6e; Figure S24). The more serious tumor injury in the RuSSRu+Light group was further proved by H&E staining and Ki67 immunofluorescence staining as compared with the results from other groups (Figure 6f,g).

To evaluate the biosafety of RuSSRu, we performed a comprehensive toxicity assessment during and after RuSSRu administration. During a 28‐day treatment, no obvious weight loss was found in each group (Figure 6a). No significant physiological morphology alterations of the mice treated with RuSSRu were observed in the major organs, including heart, liver, lung, kidney, and spleen, indicating its good bio‐safety in the body circulation (Figure 6h,i; Figure S25). Besides, the biochemical analysis of serum reflecting liver function (AST and ALT) (Figure 6j), kidney function (UREA and CREA) (Figure 6k) and heart function (CK‐MB and LDH) (Figure S25), revealed no marked abnormalities. Overall, the results demonstrated negligible systemic toxicity of RuSSRu in MC38 tumor‐bearing mice.

### 2.6 The Potential Biological Mechanism of RuSSRu‐Mediated Disulfidptosis in Tumor

**FIGURE 7:**
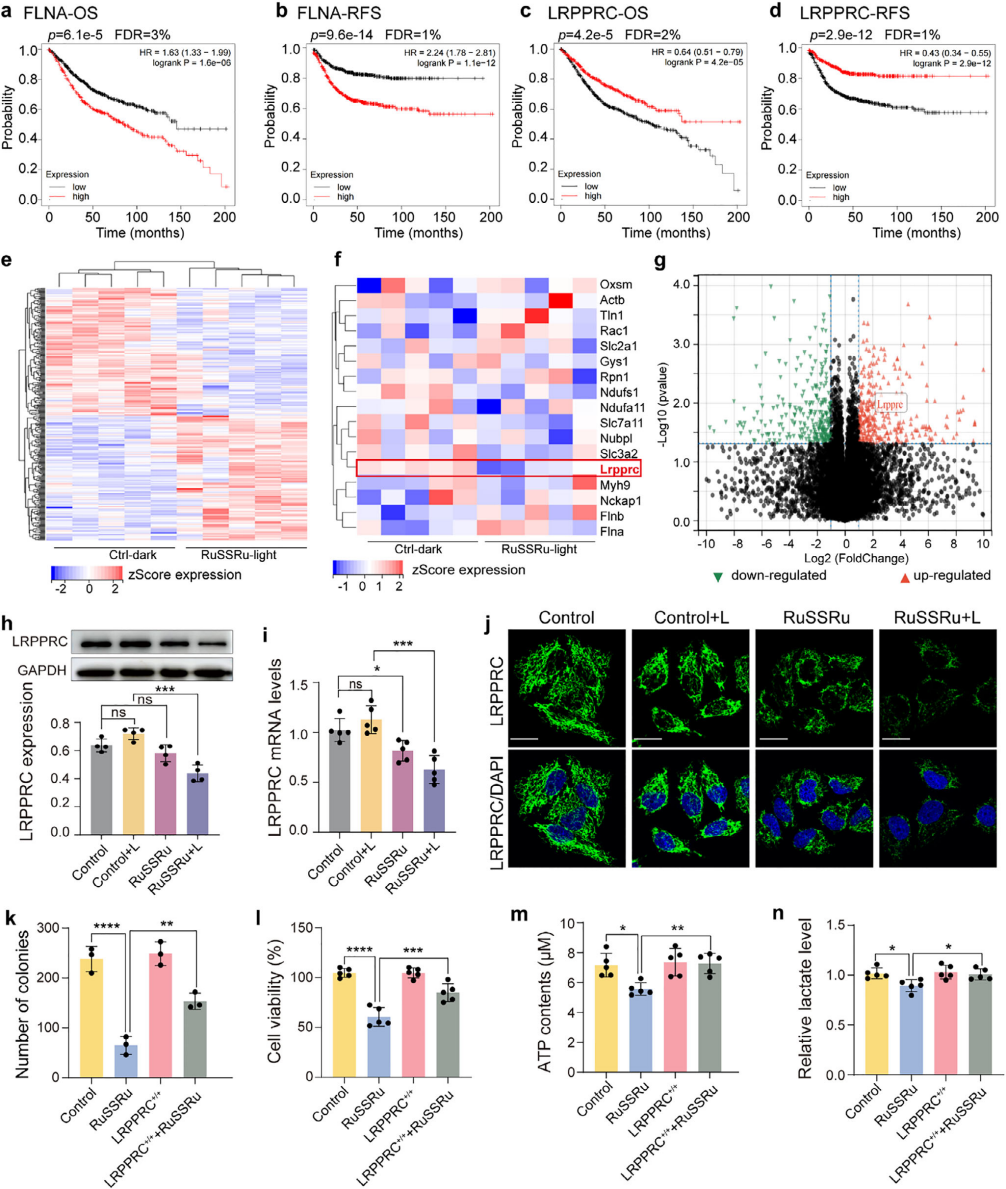
Exploring and validation of the mechanism of RuSSRu‐mediated disulfidptosis. (a,b) Kaplan–Meier analysis of OS and RFS based on FLNA mRNA levels using the KM‐plotter colon cancer database (http://kmplot.com/analysis). (c,d) Kaplan–Meier analysis of OS and RFS based on LRPPRC mRNA. (e) Cluster gram heat map of whole‐genome RNA‐sequencing data. (f) Heat map of disulfidptosis‐associated genes based on the RNA‐seq. (g) Volcano plots of total RNA‐sequencing showing DEGs among groups. (h) Western blotting of the expression of LRPPRC protein in MC38 cells, *n* = 4. (i) The mRNA levels of LRPPRC in MC38 cells, *n* = 5. (j) Representative immunofluorescent images of LRPPRC expression in MC38 cells. Scale bar = 20 µm. (k) Number of cell colonies (*n* = 3). (l) Cell viability of RuSSRu in wild‐type and LRPPRC overexpression MC38 cells (*n* = 5). (m) ATP contents (*n* = 5). (n) The lactate levels (*n* = 5). Error bar indicates Standard Deviation (SD). ^*^ *P* < 0.05, ^**^ *P* < 0.01, ^***^ *P* < 0.001, ^****^ *P* < 0.0001.

The bioinformatic analysis workflow is shown in Figure S26. Firstly, we analyzed the association between colon cancer patients and the expression of disulfidptosis‐related key differential genes from the Kaplan‐Meier plotter database (http://www.kmplot.com). The analysis showed that the expressions of 10 genes (including TLN1, MYH9, SLC2A1, NCKAP1, FLNB, SLC3A2, FLNA, NDUFA11, SLC7A11, and LRPPRC) are associated with the OS (overall survival) and RFS (recurrence‐free survival) (*P* < 0.05) of colon cancer patients (Figures S27 and S28). When the identification FDR threshold is < 5%, only the correlations of Filamin A (FLNA) and Leucine‐rich PPR motif‐containing protein (LRPPRC) are significant. The FLNA is considered a positively correlated risk factor (OR > 1) while LRPPRC is a negatively correlated risk factor (OR < 1) (Figure 7a–d).

To further explore the specific mechanism of RuSSRu‐induced disulfidptosis in colon cancer, genome‐wide RNA sequencing (RNA‐seq) from murine colon tumors in different groups was carried out. Compared with the control group, the downregulation and upregulation of related gene expression in RuSSRu+Light group are clear from the differential gene cluster heatmap (Figure 7e). Following the identification of genes implicated in disulfidptosis, subsequent heatmap analysis revealed the most significant differential expression of LRPPRC between control and RuSSRu‐Light groups (Figure 7f). The volcano plot also displayed that increased (172 genes) and decreased (177 genes) mRNA expression with a corrected *P*‐value of 0.05 and |LogFC| > 2 between two groups (Figure 7g). Interestingly, LRPPRC was obtained by taking the intersection of the key genes set above and disulfidptosis‐related genes (Figure 7g). Functional enrichment analysis based on KEGG pathway and GO pathway was performed to further elucidate the underlying function of differentially expressed genes (DEGs) (Figure S29).

To verify the role of LRPPRC‐mediated disulfidptosis, we examined the mRNA and protein expression levels of LRPPRC under different conditions in both MC38 cells and a murine tumor model. Immunoblot and immunofluorescence analyses revealed that LRPPRC expression was markedly reduced in RuSSRu+Light‐treated cells compared to the control group (Figure 7h,j). Consistently, LRPPRC mRNA levels also showed a significant decrease (Figure 7i). Consistently, LRPPRC was downregulated when colon tumors received the RuSSRu combined with PDT treatment (Figure S30). Given the important role of SLC7A11 in disulfidptosis, we also observed its expression at both the mRNA and protein levels (Figure S31). These results suggested that RuSSRu‐mediated disulfidptosis could be regulated by LRPPRC.

To directly verify whether LRPPRC plays a critical role in RuSSRu‐induced disulfidptosis, the overexpression plasmid was used to observe the effect of LRPPRC. We first investigated cell viability and clone formation changes of cells among different groups. As LRPPRC could positively regulate the cell's state, its overexpression can significantly improve the cytotoxicity induced by disulfidptosis. After RuSSRu treatment, the number and viability of cells with overexpressed LRPPRC were significantly improved (Figure 7k,l). Furthermore, the hallmarks of disulfidptosis were then assessed. Compared to wild‐type cells, ATP levels were higher in cells overexpressing LRPPRC when treated with RuSSRu (Figure 7m). And the lactate value, which is a key indicator of glucose metabolism in the tumor microenvironment, was significantly increased (Figure 7n). This suggests that compared to wild‐type cells, the glycometabolism in LRPPRC‐overexpressing cells was improved after stimulation by RuSSRu. Therefore, disulfidptosis induced by RuSSRu was mediated by LRPPRC.

In this work, a dinuclear ruthenium complex, RuSSRu, bridged by a disulfide bond, was designed to induce photoactivated disulfidptosis for highly efficient colorectal cancer treatment. Under light stimulation, the lysosome‐targeting RuSSRu generates ROS, which compromise lysosomal membrane permeability and induce lysosomal damage, thereby activating apoptotic pathways. Concurrently, the ROS oxidize NADPH, weakening its protective capacity against disulfide‐induced toxicity. Once RuSSRu enters the cytoplasm and accelerates the intracellular accumulation of disulfide bonds, it ultimately triggers disulfidptosis. This process, in conjunction with lysosomal damage‐induced apoptosis, synergistically accelerates the death of tumor cells.

Ru(II) complexes exhibit a d6 octahedral structure with ligands arranged in a 3D configuration. Due to their excellent photophysical properties and ROS generation ability, they have been widely used in the development of metal‐based anticancer drugs [20]. Disulfidptosis generally occurs under conditions of oxidative stress, when the cell produces large amounts of ROS, the sulfhydryl (─SH) groups among proteins are oxidized to disulfide bonds (─S─S─). These aberrant disulfide bonds lead to protein misfolding and functional abnormality, thereby resulting in organelle dysfunction and morphological changes and ultimately causing cell death. The reduced coenzyme NADPH can resist cellular disulfide toxicity due to its reducing property, and thus plays a vital role in the malignant proliferation of tumor cells. Once NADPH is oxidized beyond a threshold level, the excessive accumulation of disulfides within the cell will trigger cellular disulfidptosis.

Through screening in human colon cancer patients and sequencing animal models, LRPPRC was identified as a potential key molecule in RuSSRu‐mediated disulfidptosis. LRPPRC plays a pivotal role in maintaining mitochondrial function and mitigating oxidative stress. During disulfidptosis, a decrease in LRPPRC expression may cause mitochondrial dysfunction, weaken the cell's ability to manage oxidative stress and protein misfolding, and lead to programmed cell death. LRPPRC also plays a role in regulating the interaction of the cytoskeleton with a variety of cellular processes [38, 39]. This is in accordance with our results of sequencing analysis and validation. For in vivo and in vitro experiments, RuSSRu treatment inhibited tumor cell proliferation and concomitant to downregulated LRPPRC expression, which promotes the disulfidptotic response.

Although disulfidptosis is a distinct form of cell death, it may exhibit certain crosstalk and interactions with other programmed cell death, such as apoptosis and necrosis. Both disulfidptosis and apoptosis were observed in this study, which work synergistically to induce tumor cell ablation. The introduction of the disulfidptosis mechanism offers a novel perspective, enhancing our understanding of the anticancer properties of ruthenium‐based photosensitizers.

## 3 Conclusion

In conclusion, we developed RuSSRu, a dinuclear ruthenium complex capable of generating ROS to induce lysosomal damage and oxidize NADPH. Upon release into the cytoplasm, the disulfide bond in the complex exacerbates disulfide stress, leading to cytoskeletal collapse and glucose metabolism disruption, ultimately triggering disulfidptosis. When combined with lysosomal damage‐induced apoptosis, this synergistic mechanism accelerates tumor cell death. The approach demonstrates high efficacy and favorable biosafety in colorectal cancer treatment. Our work thus presents an innovative therapeutic strategy by simultaneously inducing apoptosis and disulfidptosis for enhanced cancer treatment.

## Conflicts of Interest

The authors declare no conflict of interest.

## Supporting information




**Supporting File**: advs74218‐sup‐0001‐SuppMat.docx.

## Data Availability

The data that support the findings of this study are available in the supplementary material of this article.
