# Supplementary material for: Disulfide‐Bridged Ru Complex–Mediated Photo‐Disulfidptosis for Colorectal Cancer Therapy
Source: Adv Sci (Weinh). 2026 Feb 16;13(21):e16046. doi: 10.1002/advs.202516046 (PMC13073263; doi:10.1002/advs.202516046)
Supplement: Supplementary file 1 — Supporting File: advs74218‐sup‐0001‐SuppMat.docx. [file ADVS-13-e16046-s001.docx]

**Disulfide-Bridged Ru Complex–Mediated Photo-Disulfidptosis for Colorectal Cancer Therapy**

Simeng He, ^#[a]^ Wendong Jin, ^#[b]^ Jiaojiao Pang, ^#[a]^ Xiaoting Gong, ^[c]^ Shixian Cao, ^[b]^ Duo Mao, *^[d]^ Yuguo Chen, *^[a]^ Kang-Nan Wang, *^[a], [b]^ and Bin Liu, *^[c]^

[a] Dr. S. He, Dr. J. Pang, Prof. Y. Chen, and Prof. K.-N Wang
Department of Emergency Medicine, Qilu Hospital of Shandong University, Jinan 250012 (China), Y. Chen, E-mail: [chen919085@sdu.edu.cn](mailto:chen919085@sdu.edu.cn)

[b] Dr. W. Jin, Dr. S. Cao, and Prof. K.-N Wang
State Key Laboratory of Crystal Materials, Shandong University, Jinan 250100 (China). K.-N Wang, E-mail: [wangkn@sdu.edu.cn](mailto:wangkn@sdu.edu.cn)

[c] Dr. X. Gong, Prof. B. Liu, Department of Chemical and Biomolecular Engineering, National University of Singapore, Singapore 117585 (Singapore). B. Liu, E-mail: [cheliub@nus.edu.sq](mailto:cheliub@nus.edu.sq)

[d] Prof. D. Mao
Institute of Precision Medicine, The First Affiliated Hospital of Sun Yat-Sen University, Sun Yat-Sen University, Guangzhou 510080, (China). Prof. D. Mao E-mail: [maod6@mail.sysu.edu.cn](mailto:maod6@mail.sysu.edu.cn)

**Materials and methods**

**Materials and measurements**

All materials were purchased from commercial sources. Solvents were purified by standard procedures. NMR spectra were recorded on a Bruker Advance 400 MHz or Bruker AVIII 500 MHz (Germany). Shifts were referenced relative to the internal solvent signals. ESI-MS spectra were recorded on a Thermo Finnigan LCQ DECA XP spectrometer (USA). The quoted m/z values represented the major peaks in the isotopic distribution. UV/Vis spectra were recorded on a Varian Cary 300 spectrophotometer (USA). Steady-state emission spectra were performed on an Edinburgh FLS 920 spectrometer (UK). The diameter of the probes was determined by dynamic light scattering (DLS) on a Zetasizer Nano ZS (Malvern Instruments Ltd.). The morphology and size of the samples were analyzed using transmission electron microscopy (TEM) on a JEOL JEM-2100 (Japan) operated at 200 kV. For STED imaging, both confocal and STED images were collected on the Leica TCS SP8 STED 3X Microscope with a 63u oil-immersion objective lens. For observing drug distribution, an optical imaging system (IVIS Spectrum, USA) for living small animals. For two-photon irradiation, a diode laser (808 nm, Hi-Tech Optoelectronics Co., Ltd. Beijing, China) was used.

**Synthesis and characterization**

**Scheme S1.** Synthesis route of the compound RuSSRu.

**Synthetic route of SS** (Scheme 1)

2-((2-Carboxybenzyl)thio)benzoic acid (2.88 g, 10 mmol) was dissolved with 20 mL of SOCl_2_, and the mixture was stirred under 60℃ for 2 hours. After cooling down to room temperature, the solvent was evaporated and the residue was added to the fresh dry dichloromethane. (4'-Methyl-[2,2'-bipyridin]-4-yl) methanol (2.2 g, 11 mmol) was added to the mixture slowly and stirred under room temperature overnight. The solvent was evaporated and purified by silica gel column chromatography using dichloromethane/hexane (V: V = 1:5) as eluent to yield SS as white powders (3.4 g, 52% yield). ^1^H NMR (CDCl_3_-*d*, 400 MHz) δ (ppm): 8.46 (d, *J* = 9.2 Hz, 2H), 8.14 – 8.04 (m, 4H), 7.99 – 7.94 (m, 2H), 7.89-7.56 (m, 2H), 4.18 (s, 3H). ^13^C NMR (CDCl_3_, 101 MHz) δ (ppm): 164.90, 155.76, 154.46, 148.53, 148.00, 147.23, 144.60, 139.72, 132.47, 130.68, 125.71, 124.96, 124.64, 123.94, 121.07, 121.04, 118.68, 64.44, 28.67, 20.20. HRMS (mass m/z): calcd for [M+H] ^+^: 671.1781, found: 671.1786.

**Synthetic route of RuSSRu**

SS (670 mg, 1 mmol) and [ruthenium(2,2′-bipyridine)_2_Cl_2_] (400 mg, 1 mmol) was dissolved with 20 mL of methanol, and then the mixture was stirred under 85℃ for 12 hours. After cooling down to room temperature, the solvent was evaporated and the residue was dissolved with a solution of ammonium hexafluorophosphate (652 mg, 4 mmol, dissolved in 40 mL of water). The mixture was refluxed overnight. After cooling down to room temperature, 20 mL of dichloromethane was added to the solution and the organic layer was separated, whereas the aqueous layer was extracted two times with 50 mL of dichloromethane. Then, combined extracts were dried over Na_2_SO_4_. The solvent was evaporated and the residue was purified by silica gel column chromatography using dichloromethane/methanol (V: V = 100:1) as eluent to yield RuSSRu as pile yellow powders (457 mg, 26% yield). ^1^H NMR (DMSO-*d_6_*, 500 MHz) δ (ppm): 8.96 (s, 2H), 8.84 (d, *J* = 8.2 Hz, 8H), 8.75 (s, 2H), 8.23 (d, *J* = 7.9 Hz, 2H), 8.17 (t, *J* = 7.9 Hz, 8H), 7.80 – 7.71 (m, 10H), 7.68 – 7.61 (m, 6H), 7.56 (dt, *J* = 12.8, 6.0 Hz, 10H), 7.41 (t, *J* = 7.3 Hz, 4H), 5.64 (s, 4H), 2.54 (s, 6H). ^13^C NMR (CDCl_3_,101 MHz) δ (ppm): 165.62, 157.09, 157.06, 157.02, 151.69, 151.64, 139.58, 138.32, 134.62, 132.50, 129.31, 128.34, 126.93, 126.76, 126.17, 125.78, 125.71, 124.91, 123.02, 64.90, 21.13. HRMS (mass m/z): calcd for [M-PF_6_]^+^: 1933.1487, found:1933.1489.

**Calculating quantum yield (QY) of RuSSRu**

We utilized a relative strategy to measure the quantum yield (QY) of the two compounds in our work^1,2^. The QY of the two compounds were measured using a standard dye R6G as the reference. The calculation equation was:

$$\mathrm{QY}_{sam}=\mathrm{QY}_{ref}\times\frac{K_{sam}}{K_{ref}}\times\left( \frac{S_{sam}}{n_{ref}} \right)^{2}$$

Where QY_sam_ is the QY of the RuSSRu, QY_ref_ is the QY of R6G (QY= 0.94 in ethyl alcoho)^3^, K_sam_ and K_ref_ are the slopes obtained by linear fitting of the integrated emission spectra of RuSSRu (535-750 nm) and R6G (475-675 nm) against the absorbance at 450 nm, respectively, n_sam_ and n_ref_ are the refractive indices of their respective solvents (water: 1.33; and dichloroethane: 1.42; dimethyl sulphoxide:1.47; methanol:1.32; ethyl alcohol:1.36; ethyl alcohol:1.36).

**Two-photon fluorescence spectroscopy acquisition**

We utilized the following strategy to measure the two-photon absorption cross-sections in our work^4^. The two-photon fluorescence test was performed by a Ti: Sapphire Mira 900-F femtosecond laser with a pulsed laser light source of 200 pulses and a repetition rate of 76 MHz, and the data was recorded with a Spectro Pro 300i spectrometer. The standard dye R6G was used as a reference to measure the two-photon absorption cross-sections of the two compounds. The calculation equation was estimated as:

$$\delta_{sam}=\delta_{r}\times\frac{\Phi_{ref}c_{ref}n_{ref}F_{sam}}{\Phi_{sam}c_{sam}n_{sam}F_{ref}}$$

where δ_sam_ and δ_ref_ are the two-photon absorption cross-section of sample (RuSSRu) and reference (R6G), respectively. c_sam_ and c_ref_ are the concentration of sample and reference (10 μM). n_sam_ and n_ref_ are the refractive indices of their respective solvents (water: 1.33, ethyl alcohol:1.36). F_sam_ and F_ref_ are the fluorescence integration intensity of two-photon excitation.

**Transmission electron microscopy**

The morphology and size of the samples were analyzed using transmission electron microscopy (TEM) on a JEOL JEM-2100 (Japan) operated at 200 kV. The solid RuSSRu compound was directly dissolved in ultrapure water to prepare sample suspensions. RuSSRu was dispersed in water and then, a drop of the sample suspension was placed onto a carbon-coated copper grid, and allowed to dry at room temperature. Excess liquid was removed using filter paper, and the sample was further dried under vacuum before imaging. To enhance contrast, samples were negatively stained with 2% uranyl acetate for 1 min, followed by air-drying. Images were acquired using a Gatan CCD camera, and particle sizes were analyzed using Digital Micrograph 3.0 software. The concentration of RuSSRu used for the test is 10 μM and 1 mM, respectively.

**Dynamic light scattering**

The particle size distribution of RuSSRu was measured using dynamic light scattering (DLS) on a Zetasizer Nano ZS (Malvern Instruments, UK) at 25°C. The samples were dispersed in pH = 7.4 PBS buffer and filtered through a 0.22 μm membrane to remove large aggregates. Measurements were performed in a cuvette with a detection angle of 173° (backscatter mode). The hydrodynamic diameter was determined based on the intensity-weighted size distribution using the cumulant analysis method. Each sample was measured at least 3 times, and the average particle size along with the polydispersity index (PDI) was reported. The instrument was calibrated with standard polystyrene nanoparticles before measurement. The concentration of RuSSRu used for the test is 1 mM.

**Zeta potential**

The zeta potential of RuSSRu was measured using a Zetasizer Nano ZS (Malvern Instruments, UK) at 25°C. The samples were dispersed in an appropriate buffer solution (pH = 7.4 PBS buffer) and filtered through a 0.22 μm membrane to remove large aggregates. Measurements were performed in a folded capillary cell with an applied electric field of 150 V. The zeta potential was determined based on the electrophoretic mobility using the Helmholtz-Smoluchowski equation. The average value along with the standard deviation (Mean ± SD) was reported. The instrument was calibrated with polystyrene latex standards before measurement. The concentration of RuSSRu used for the test is 10 μM and 1 mM, respectively.

**Optical properties**

The UV-Vis absorption spectra and fluorescence emission spectra were carried out in PBS or in solvents with different polarities.

**Photoactivated ability of RuSSRu**

We utilized the following strategy to measure the ROS in our work^5^. Hydroxyphenyl fluorescein (HPF, Sigma) and Dihydrorhodamine 123 (DHR123, Sigma) were utilized to detect the generation of OH^−•^ and O_2_^−•^ of the PSs in an aqueous solution under white light irradiation (30 mW/cm^2^), respectively. In brief, 2 μL of [Ru(bpy)_3_]Cl_2_, RuOH or RuSSRu (10 mM), 20 μL of SS (1 mM), 4 μL RB (5 mM), or 4 μL Ce6 (5 mM) was diluted in 2 mL PBS, and then 2 μL 5 mM HPF or DHR123 were mixed in quartz cuvette. The fluorescence of HPF and DHR123 induced by different compounds OH^−•^ and O_2_^−•^ under irradiation of white light (30 mW/cm^2^) were measured at different time intervals. Fluorescence was measured at 490/515 nm and 473/526 nm for HPF and DHR123, respectively, using a fluorescence spectrometer.

DCFH-DA (Sigma) was utilized to detect the total ROS generation of the PSs in an aqueous solution under white light irradiation (30 mW/cm^2^). In brief, 0.5 mL of DCFH-DA (1 mM) and 2 mL of NaOH (10 mM) in water were mixed and stirred at room temperature for 30 min. Hydrolysate DCFH-DA was then neutralized by 10 mL of PBS (pH = 7.4, 0.01 M). 2 μL of [Ru(bpy)_3_]Cl_2_, RuOH or RuSSRu (10 mM), 20 μL of SS (1 mM), 4 μL of RB (5 mM), or 4 μL Ce6 (5 mM) was diluted in 2 mL 40 μM DCFH hydrolysate were mixed and transferred to a quartz cuvette. The fluorescence of DCFH induced by different probes under irradiation of white light (30 mW/cm^2^) was measured at different time intervals. Fluorescence was measured at 488/525 nm using a fluorescence spectrometer. 9,10-anthracenediyl-bis(methylene) dimalonic acid (ABDA, Sigma) was utilized to detect the generation of ^1^O_2_ of the PSs in an aqueous solution under white light irradiation (30 mW/cm^2^). In brief, 2 μL of [Ru(bpy)_3_]Cl_2_, RuOH or RuSSRu (10 mM), 20 μL of SS (1 mM), 4 μL RB (5 mM) or 4 μL Ce6 (5 mM) was diluted in 2 mL PBS, and then 4.1 μL of 4.87 mM ABDA were mixed and transferred to a quartz cuvette. The absorption spectra of ABDA were monitored in a range of 320-420 nm after the solution was irradiated by irradiation of white light. The decreased absorbance of ABDA at 378 nm was recorded to indicate the generation rates of singlet oxygen.

**Cell culture and co-localization analysis**

Cells were cultured by DMEM medium and in the standard cell incubator. Cells were seeded on confocal dish at a density of 5×10^4^ cells/well and incubated for 12 h. Removing the culture medium, cells were cultured with RuSSRu (2 μM) for 6 h under different conditions, then the prepared Lyso-Tracker^TM^ Deep Red staining solution (200 nM) and Mito-Tracker^TM^ Deep Red (200 nM) was added and incubated for 20 min at 37°C. Subsequently, cells were investigated by confocal laser endomicroscopy (Leica, Germany).

**Western Blotting analysis**

Proteins were extracted from heart, liver, lung, kidney tissues. The bicinchoninic acid (BCA) reagent (Beyotime, China) was used to determine protein concentrations. 20 μg samples were loaded on 6%-15% SDS-PAGE for electrophoresis and then transfer to a PVDF membrane (Millipore), and then incubated with primary antibodies for Bax (1:1000, Abcam, ab32503), Bcl-2 (1:2000, Abcam, ab182858), Cleaved-caspase 3 (1:5000, Abcam, ab214430), TFEB (1:3000, Proteintech, 13372-1-AP), LRPPRC (1:5000, Proteintech, 21175-1-AP), SLC7A11 (1:1000, CST, #12691) and GAPDH (1:5000, Proteintech, 60004-1-Ig) at 4°C overnight. After washed with TBST three times, the blots were infiltrated with appropriated secondary antibody (1:10000, Jackson, 111-035-003) for 1~2 h at room temperature. Finally, the blots were visualized by enhanced chemiluminescence system (Merck-Millipore, WBKLS0500, USA) and analyzed using ImageJ system. All the original data of western blot could be found in the Supporting Information.

**Dark and Light Toxicities**

MC38 and HCT116 cells were dissociated and resuspended at a density of 10 × 10^4^ cells/mL, then seeded into 96-well plates (100 μL/each well), and cultured overnight. Cells were then treated with various concentrations of RuOH, RuSSRu, [Ru(bpy)₃]Cl_2_, and SS for 6 h. After washing with PBS, cells were or were not exposed to light irradiation at 425 nm for 10 min (30 mW cm^-2^) and cultured for another 12 or 24 h. The cytotoxicity of RuSSRu drugs with or without light irradiation was determined *via* CCK8 assay (Beyotime, China).

**ICP-MS determination**

Briefly, the cells were plated into six-well plates and 1mL 69% HNO_3_ was added to each well to digest for 2 h. The samples were added ddH_2_O to obtain a final volume of 3 mL. Ru was added as the internal standard at a final concentration of 3 µg/L. The samples were analyzed using Agilent 5100 / ICP-MS PE300D.

**Intracellular ROS detection**

After attachment of MC38 cells incubated in six-well plates, the medium containing 2 μM RuSSRu was switched to continue incubating for 6 h. After incubation in the dark or 425 nm light for 10 min, cells were observed using the DCFH-DA fluorescent probes (Beyotime, Shanghai, China) and then collected for confocal microscope.

**NADPH levels**

NADPH levels were determined using NADP/NADPH Quantification Kit. Briefly, NADP^+^/NADPH extracts were added in the cultured six-well plates (200 μL/each well), blown gently to facilitate cell lysis, followed by centrifuged at 12000 *g* for 10 min at 4°C. Taking the supernatant 50 μL to 96-well plates and added the G6PDH working solution in the plates for incubation at 37°C for 30 min protected from light. Then, the color development solution (10 μL) was added and incubated for 10-20 min at 37°C, the absorbance of the samples was then measured.

**ATP contents**

Pre-treated MC38 cells were processed for measurement of ATP contents using a commercial kit (Beyotime S0026, China). This ATP assay kit is based on the quantitative measurement of a stable level of light by luciferase-catalyzed enzymatic reactions. Approximately 1×10^6^ cells were mixed thoroughly in 200 μL cold lysis buffer and centrifuged at 12000 *g* for 3 min at 4°C. The collected supernatant was added to 100 μL ATP assay buffer and then the ATP concentrations were calculated from the standard curve data.

**Cysteine levels**

MC38 Cells were plated in a glass bottom Petri dish (35 mm) for 24 h and were treated with RuSSRu in the dark/light. After treatment, cysteine extracts were added in the dishes and centrifuged at 11000 rpm at 4°C, the supernatant was collected and monitored by the BioTek microplate reader (600 nm).

**Lactate levels**

Approximately 2×10^6^ MC38 cells under different conditions were harvested and washed with cold PBS. 200 μL lactate assay buffer was added in the cells and homogenized quickly followed centrifuged at top speed for 5 min at 4°C. The supernatant was evaluated according to the manufacture instruction (Abcam, ab65330, Shanghai, China).

**Detection of apoptosis**

The apoptosis of cells was assessed using an PI Annexin V Apoptosis Detection kit (BD Pharmingen™, USA) following the instruction manual. Cells were collected after dissociation and rinsed with prechilled PBS after 24 h in culture. Subsequently, the precipitate was collected in 300 μL binding buffer, followed by incubation with Annexin V (5 μL, 15 min) and later with PI (5 μL, 5 min) shielded from light at room temperature. The intensity was measured using a CytoFLEX flow cytometer (Beckman Coulter, Brea, CA, USA) and analyzed by FlowJo v10 software (BD Biosciences, USA).

**Cell clone formation ability**

Cells were re-suspended in complete medium and seeded into 6-well plates with 100 cells per plate, and cultured in a 5% CO_2_ and 37°C cell culture incubator. The medium was changed every 2-3 days until 14 days. 0.5-1 mL of 4% paraformaldehyde was added to each well and fixed for 30 min, then 1 mL crystal violet dye was added and incubated for 10 min at room temperature. After washing 3-5 times, natural air drying, the 6-well plate were observed and cell clones were counted.

**Measurement of intracellular GSH and MDA detection**

Attached cells in six-well plate incubated with RuSSRu for 6 h followed by light (425 nm, 10 min), and incubated in the dark as controls. Cells were collected and washed by cold PBS solutions for 2 times, followed by disruption using sonication. Intracellular GSH and MDA levels were determined using the commercial kits according to the manufacturer’s instructions.

**Preparation of RuSSRu in vivo study**

Taking 1 mL of stock solution with a concentration of 5 mg/mL as an example, 5 mg of RuSSRu is dissolved in 100 μL of DMSO, and then 900 μL of sterile physiological saline is added and mixed evenly to obtain the stock solution. The stock solution was further diluted 5-fold with saline as a working solution (1 mg/mL RuSSRu (2% v/v DMSO)) for intravenous injection. The mice were administered with RuSSRu intravenously at a dose of 5 mg/kg and a volume of 100 μL per mouse before further imaging.

**Distribution imaging in vivo**

In vivo RuSSRu distribution imaging was evaluated in tumor-wearing C57BL/6J mice. After subcutaneously injection of 1×10^6^ MC38 cells (expressed luciferase), tumor growth was monitored until tumors grew to treatable levels (∼1500 mm^3^). 100 μL of RuSSRu (5 mg/kg) was injected into mice via tail vein, the organs and tumor tissue were harvested after 6 h. And the drug distribution was observed using an optical imaging system (IVIS Spectrum, USA) for living small animals (λ_ex/em_ = 450 nm / 470-620 nm).

**In vivo antitumor efficacy investigation (syngeneic models)**

The animal study was approved by the Medical Ethics Committee of Qilu hospital of Shandong University (No. DWLL-2024-145). Male C57BL/6J mice (6-week-old) were purchased from Charies River Laboratory Animal Co. Ltd. (license No. SCXK (Beijing) 2021-0006, Beijing, China). The mice were inoculated subcutaneously with MC38 cells (1×10^6^/mouse) under the left armpit. When the tumor volume reached 200 mm^3^ (day 7 after injection), the mice were divided into 5 groups randomly (n = 6): control-dark treated (ctrl-dark), control-light treated (ctrl-light), RuSSRu-dark treated (RuSSRu-dark), RuSSRu-light treated (RuSSRu-light), and Cisplatin. RuSSRu (5 mg/kg) was injected intratumorally twice a week and treated with two-photon irradiation (808 nm, 40 mW cm^-2^) for 10 min at 6 h after injection. Noteworthy, the introduction of two-photon irradiation can significantly enhance tissue penetration. Cisplatin was injected intraperitoneal (5 mg/kg), and 0.9% saline was considered as vehicle control. The body weight and tumor volume were recoded every 3 days. Tumor volume was calculated as V (mm^3^) = (length × width^2^)/2. Mice were excluded from the study when the tumor volume exceeded 1500 mm^3^. On day 28, mice were anesthetized and euthanized, the tumors were isolated and weighted, serum and major organs including heart, lung, liver, kidney were separated and stored at -80 °C for further analysis.

**Histological analysis**

The collected tissues were fixed with 4% paraformaldehyde for over 48 h followed by routine dehydration, transparency, wax immersion and embedding with 4 μm sections. After hematoxylin/eosin (H&E) staining, dehydrated and transparently sealed, slices were scanned by digital slice scanner (Olympus VS200).

**Immunofluorescence**

Slices or cells seeded in the confocal dish were fixed with 4% paraformaldehyde for 10 min and permeabilized with 0.1% Triton X-100 for 10 min. After blocking with 5% goat serum for 30 min at 37°C, slices or cells were incubated overnight with LRPPRC antibody (1: 200, Proteintech, 21175-1-AP), Phalloidin-iFluor 488 (1:1000, Abcam, ab176759). Then, slices were using PBS solution and the secondary antibody was incubated for 60-90 min at 37 °C in the dark, followed by DAPI incubation to labeled the nuclei.

**Assessment of organ injury markers**

Plasma alanine aminotransferase (ALT), aspartate aminotransferase (AST), urea (UREA), creatinine (CREA), creatine kinase myocardial band (CKMB), lactate dehydrogenase (LDH) levels were analyzed by an automatic chemical analyzer (BS-240 Chemistry Analyzer; Mindray, Shenzhen China).

**Quantitative real-time reverse transcriptase polymerase chain reaction**

Total RNA from tissues were isolated using TRIzol method (Invitrogen, Carlsbad, CA) following the manufacturer’s manuals. 1 μg RNA was converted into cDNA using Hifair® Ⅲ 1st Strand cDNA Synthesis Mix (Vazyme, Nanjing, China) under the standardized procedure: 25°C for 5 min, 55°C for 15 min and 85°C for 5 min. qPCR analysis was performed using ChamQ Universal SYBR qPCR Master Mix (Vazyme, Nanjing, China). Applied Biosystems 7500 was performed for pre-degeneration at 95°C for 30 s, followed by 40 thermal cycles consisting of denaturing for 10 s at 95°C and annealing for 30 s at 60°C. The 2^−ΔΔCt^ method was calculated to determine the relative mRNA expression of LRPPRC genes and the primers were: LRPPRC: forward: 5’-GACCCGCGCGTTGGC, reverse: 5’-TCCCTTTTCTCAGCAACGATGG; GAPDH: forward: 5’-TGTCTCCTGCGACTTCAACA, reverse: 5’-GGTGGTCCAGGGTTTCTTACT.

**Statistical analysis**

Analysis of data in the study was performed with one-way ANOVA with Tukey’s test through a GraphPad Prism software package (Prism 8.3.0, GraphPad software). Statistical significance was set as follows: **P* < 0.05, ***P* < 0.01, ****P* < 0.001, *****P* < 0.0001.


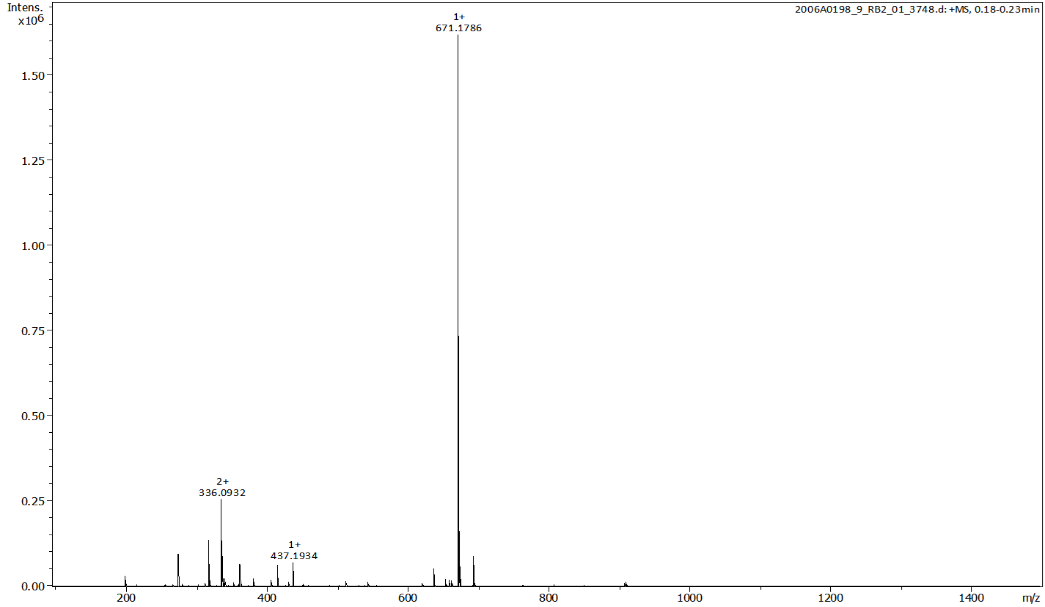


**Figure S1**. HRMS spectrum of SS.


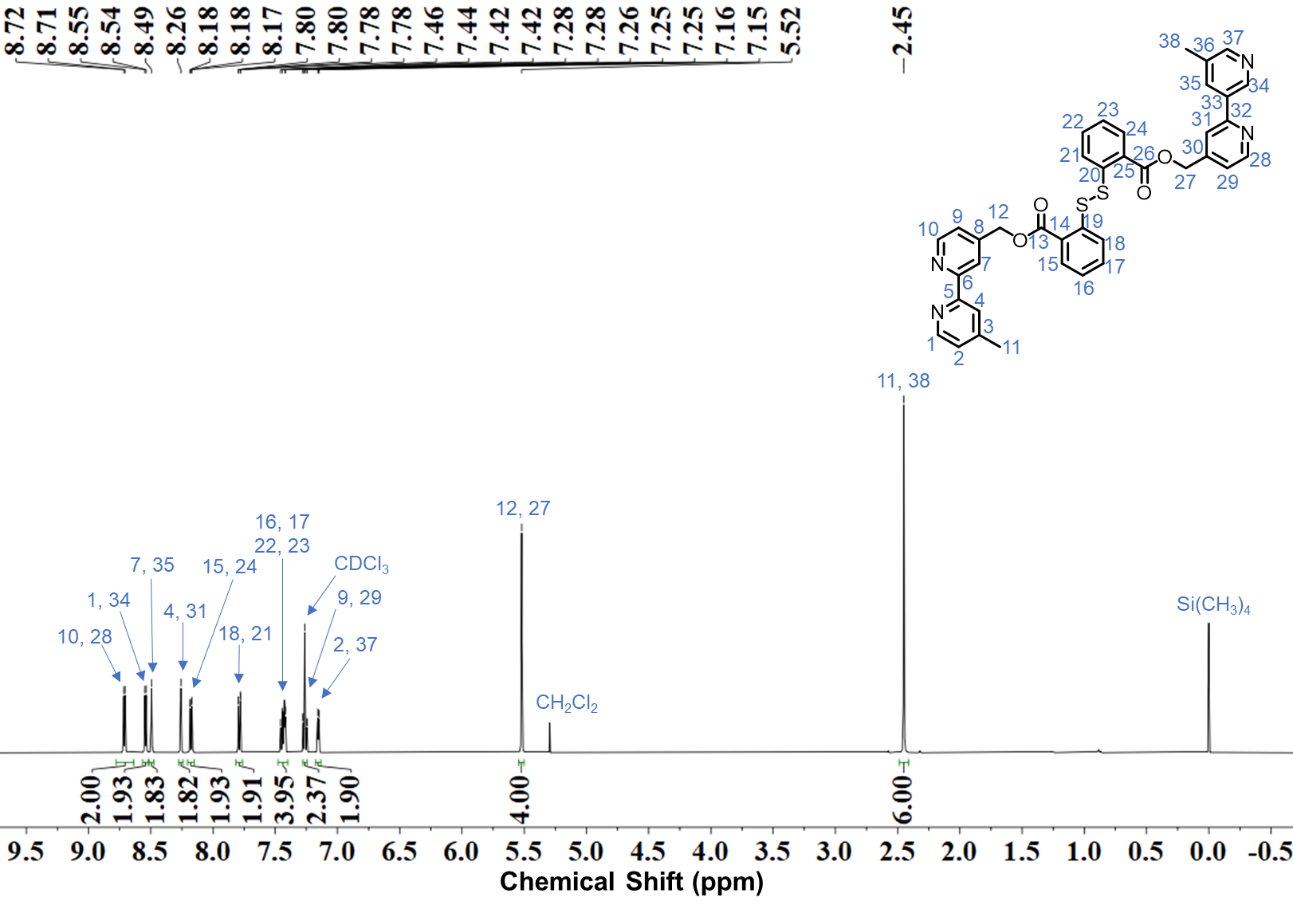


**Figure S2.** ^1^H NMR spectrum of SS.


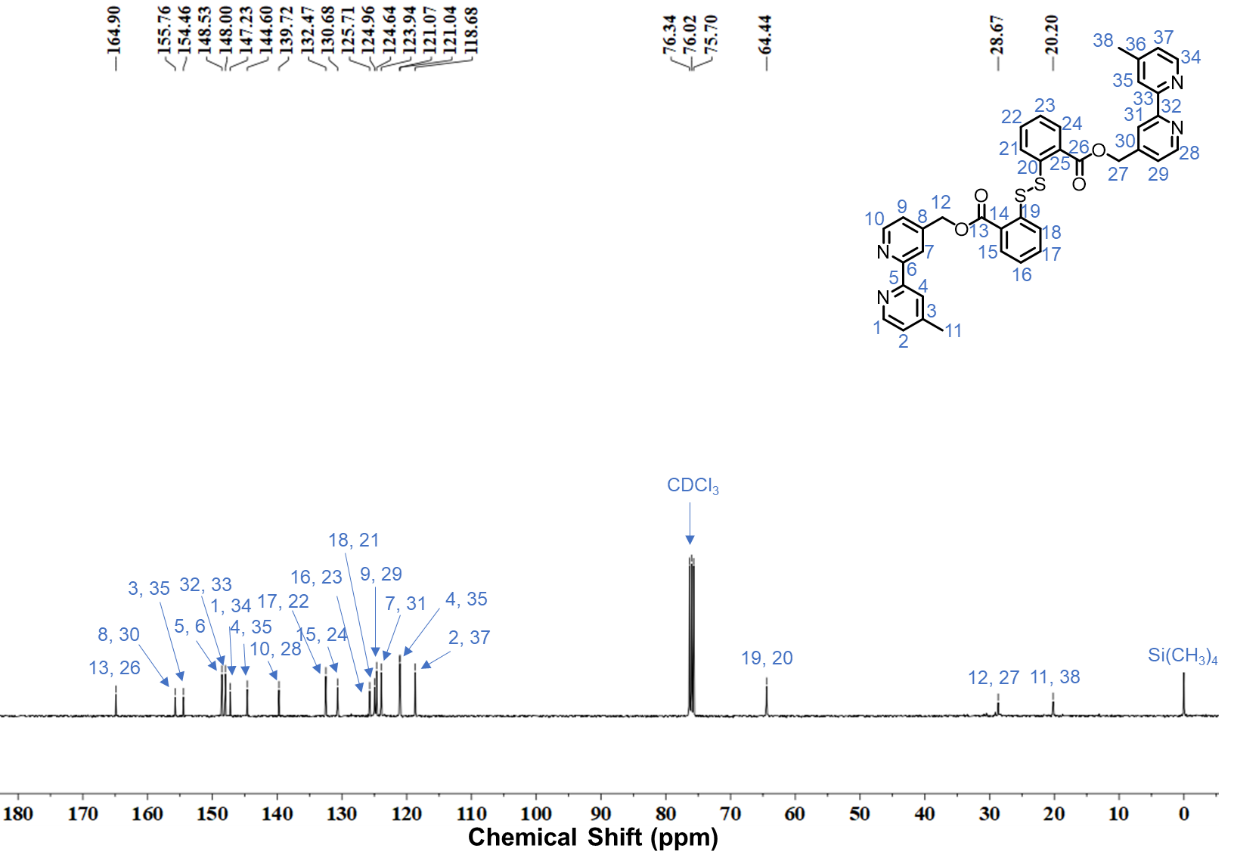


**Figure S3.** ^13^C NMR spectrum of SS.

**Figure S4.** HRMS spectrum of RuSSRu.


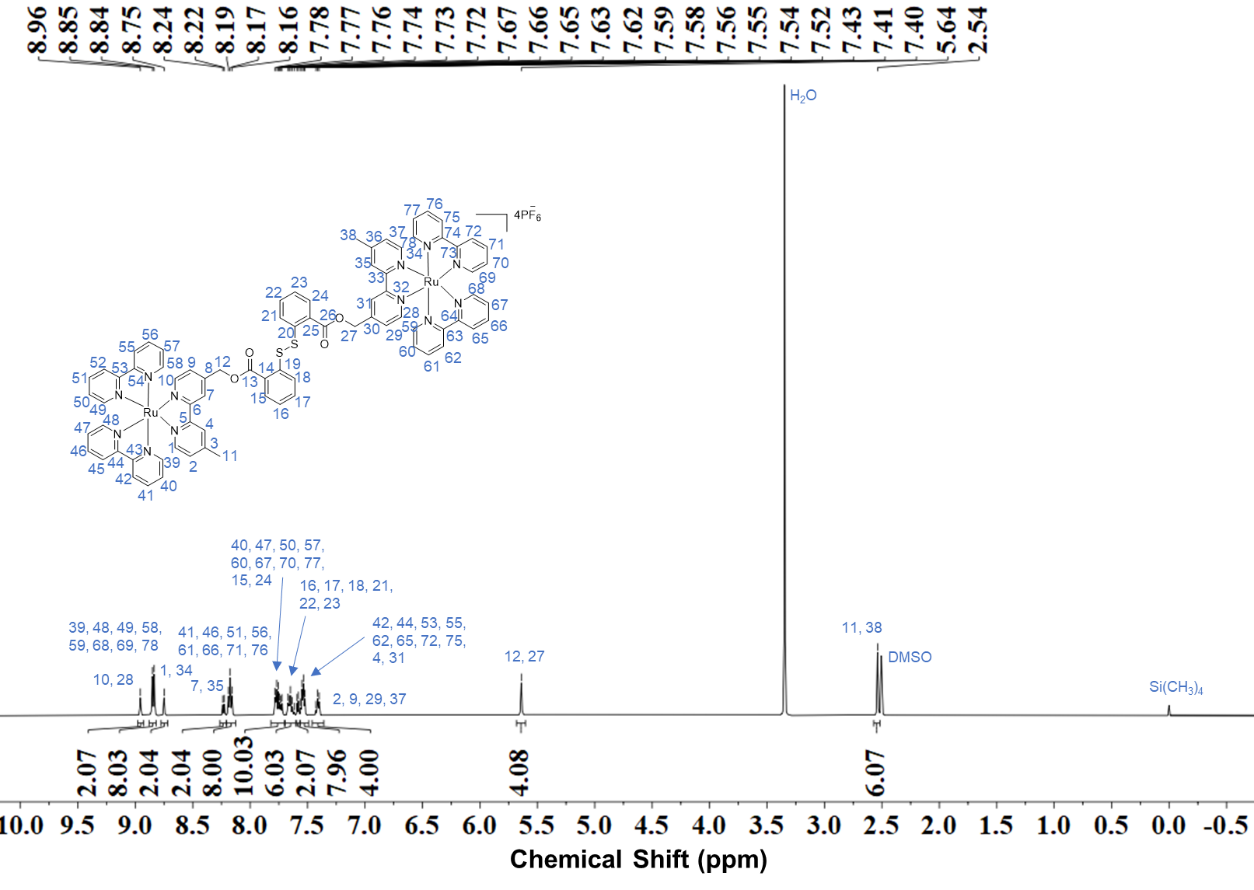


**Figure S5.** ^1^H NMR spectrum of RuSSRu.


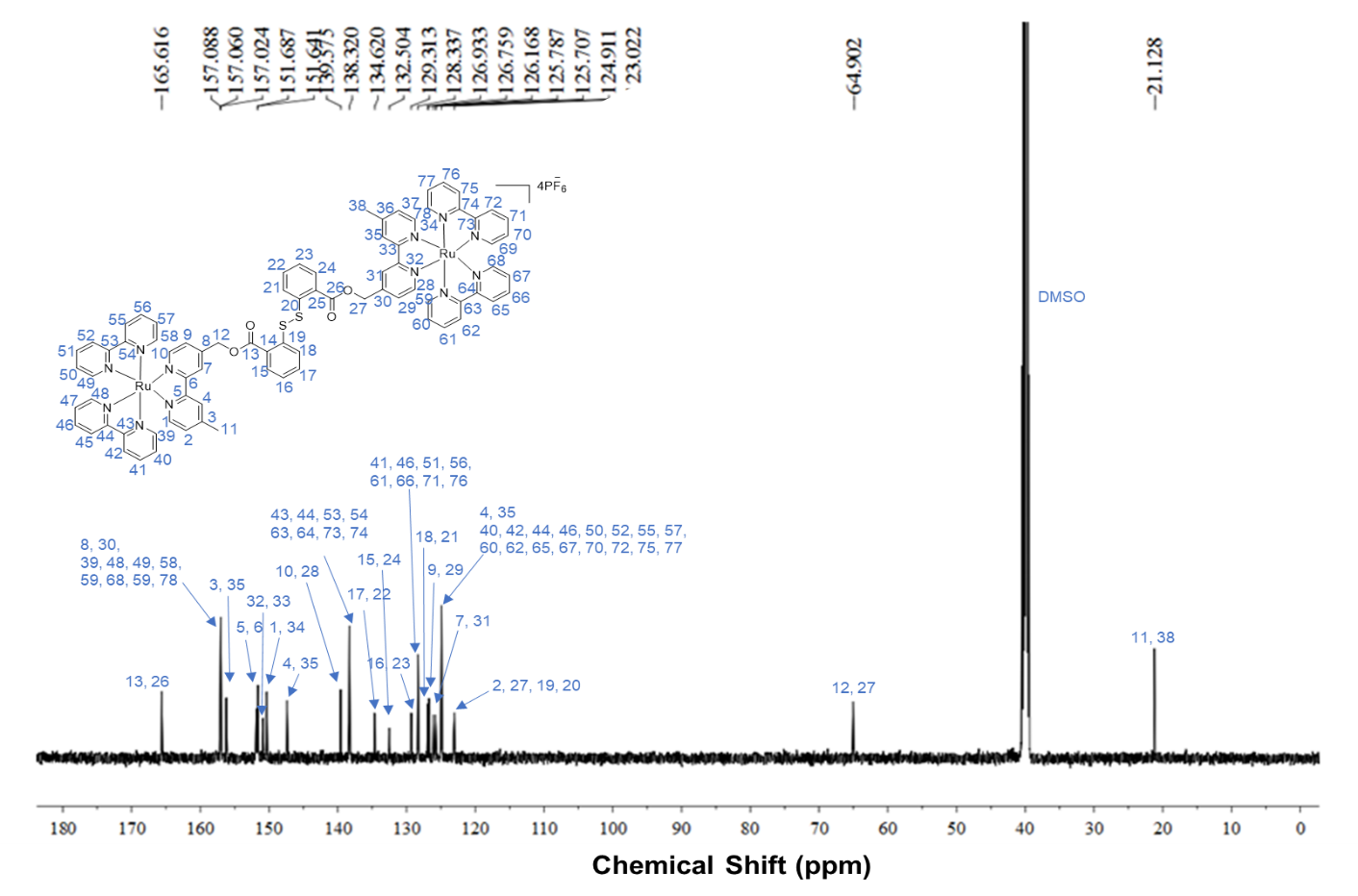


**Figure S6.** ^13^C NMR spectrum of RuSSRu.


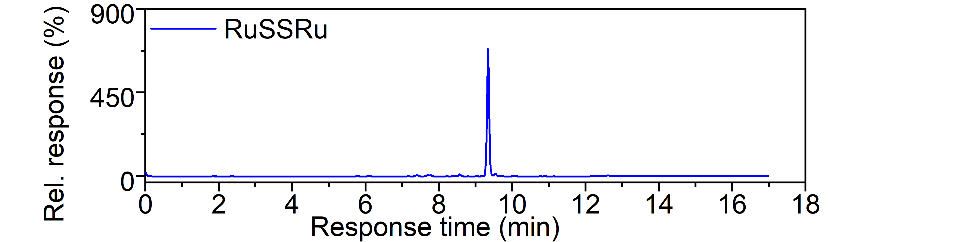


**Figure S7:** HPLC spectra of RuSSRu (mobile phase: acetonitrile/water with 0.1% TFA, detection at 254 nm).


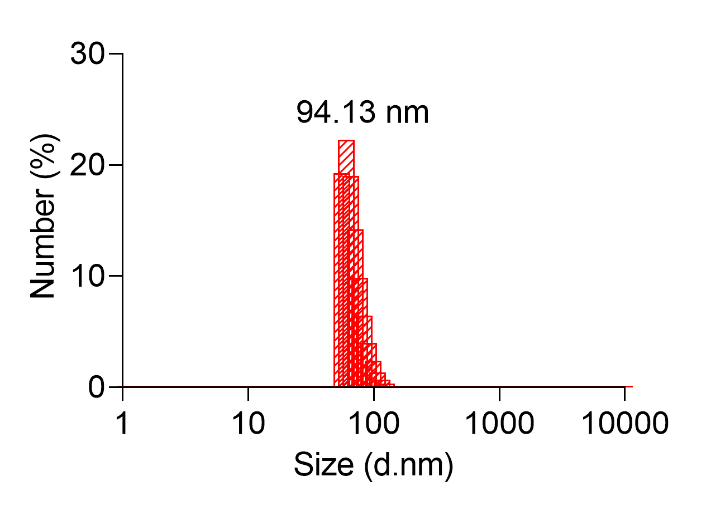


**Figure S8.** The particle size of RuSSRu in aggregation.


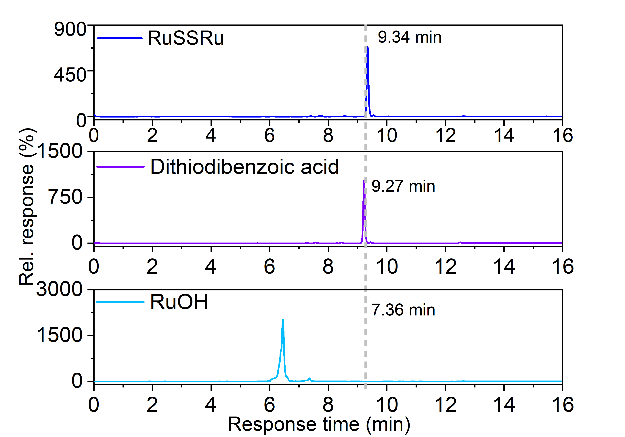


**Figure S9:** Representative HPLC spectra of RuSSRu, dithiodibenzoic acid, and RuOH recorded under identical chromatographic conditions (mobile phase: acetonitrile/water with 0.1% TFA, detection at 254 nm).


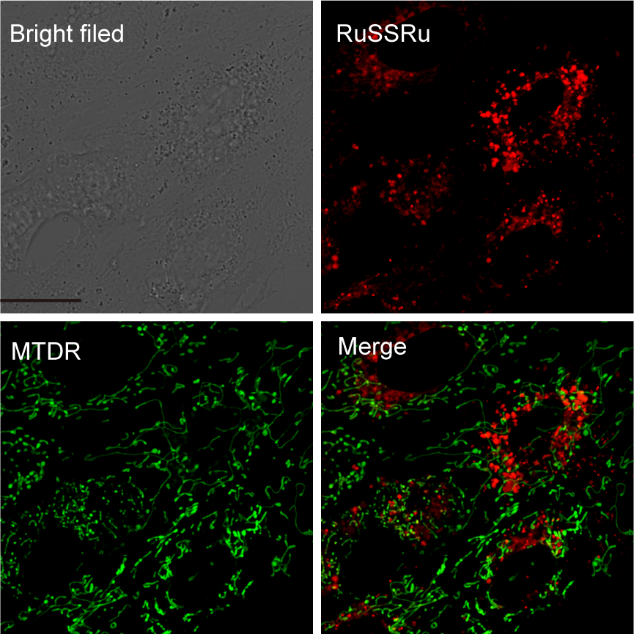


**Figure S10.** Co-localization between RuSSRu (2 μM) and mitochondrial probe MTDR (200 nM), λ_ex/em_ = 405 nm / 600 ± 20 nm for RuSSRu (red); λ_ex/em_ = 633 nm / 700 ± 10 nm for MTDR (pseudo-green). Scale bar = 20 μm.


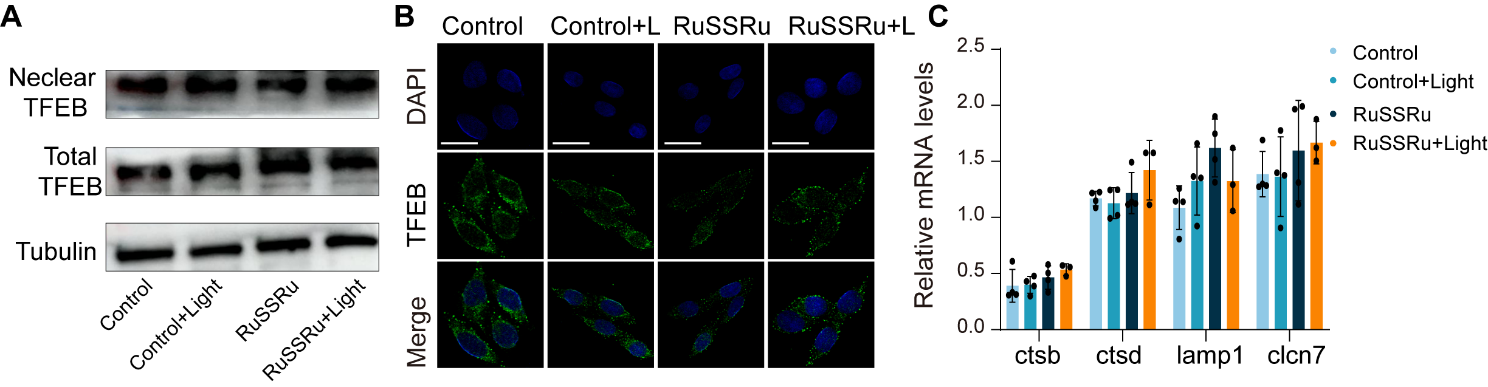


**Figure S11.** A) Western blotting of nuclear and total TFEB (Neclear TFEB, n = 7, Total TFEB, n = 8). B) Immunofluorescence analysis of TFEB intracellular localization. Scale bar = 20 μm. C) qRT-PCR was determined the relative mRNA levels of *Ctsb*, *Ctsd*, *Lamp1*, and *Clcn7*.


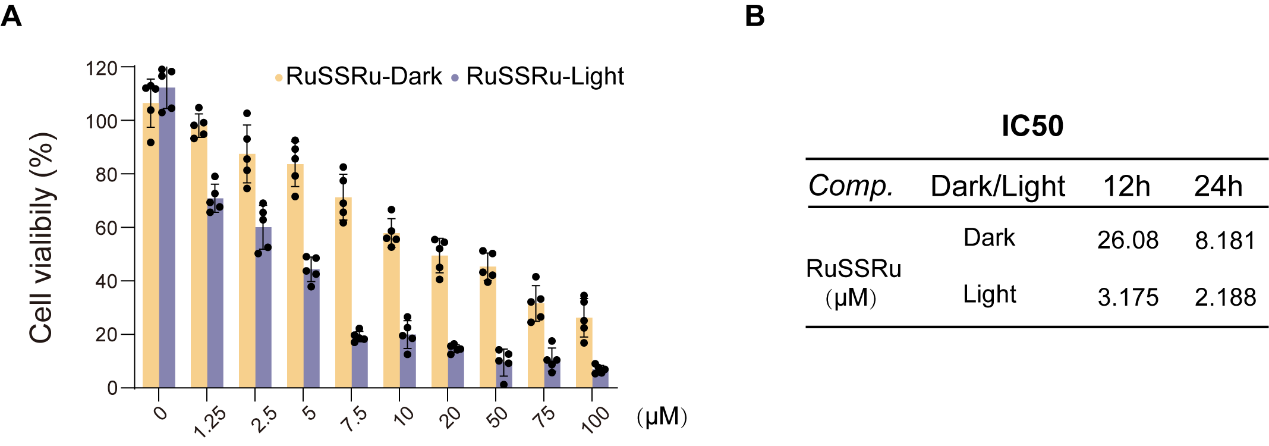


**Figure S12.** Cell viability of MC38 cells incubated with RuSSRu for 12 h with or without light irradiation (A). The IC50 of RuSSRu under different conditions (B).


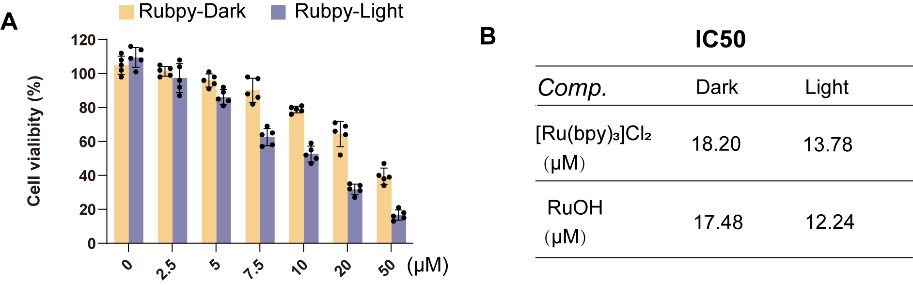


**Figure S13.** Viability of MC38 cells incubated with [Ru(bpy)₃]Cl_2_ (abbreviated as Rubpy) for 24 h (A) with or without light irradiation. (B) IC50 values of RuOH and [Ru(bpy)₃]Cl_2_ under different conditions.


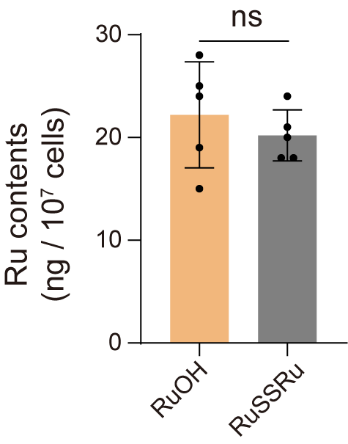


**Figure S14.** Ruthenium contents of cells after incubation with RuOH or RuSSRu for 12 h are determined by inductively coupled plasma mass spectrometry (ICP-MS).


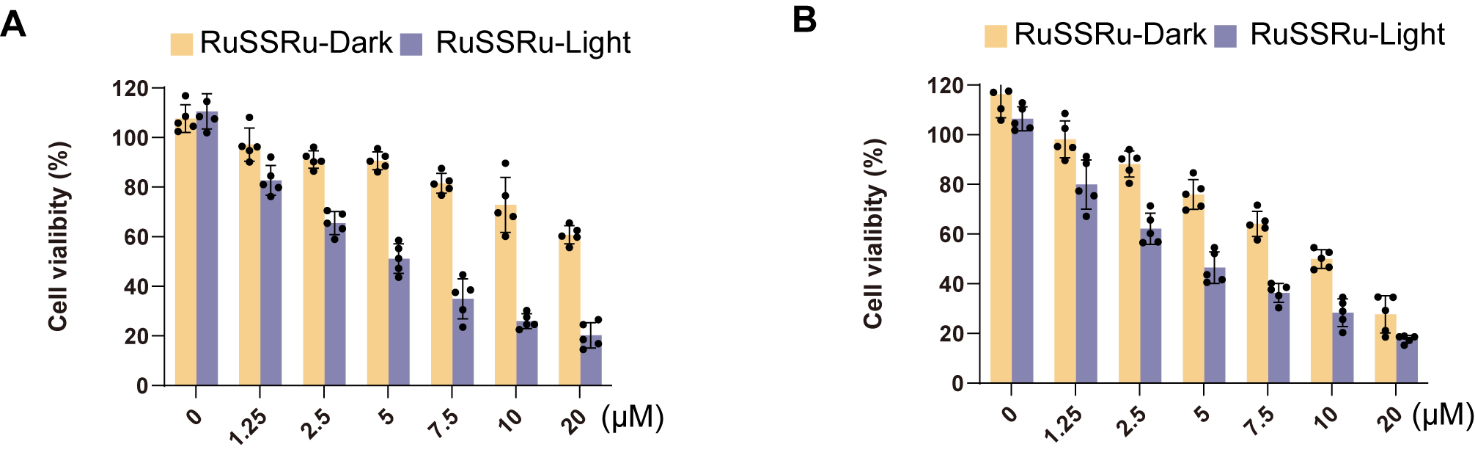


**Figure S15.** Cell viability of HCT116 cells incubated with RuSSRu for 12 h (A) and 24 h (B) with or without light irradiation.


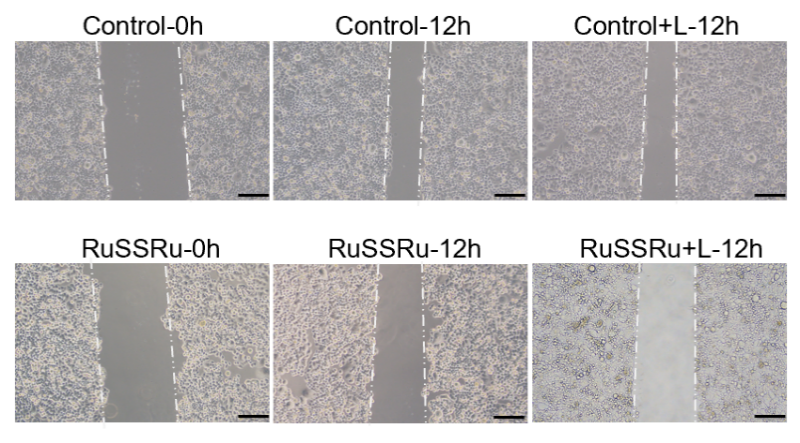


**Figure S16.** Scratch experimental results of MC38 cells, for the light stimulation group, cells were exposed to light irradiation at 425 nm for 10 min (30 mW cm-2). Scare bar = 200 μm.


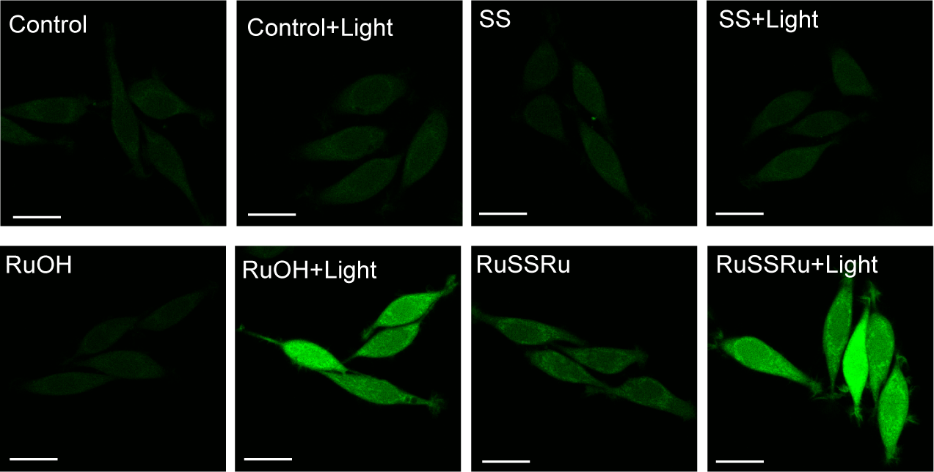


**Figure S17.** Intracellular fluorescence intensity of DCFH-DA with RuOH, SS and RuSSRu for overall ROS detection, under dark or light irradiation. For DCFH-DA, λ_ex/em_ = 488 nm /525 ± 20 nm. Scale bar = 20 μm.


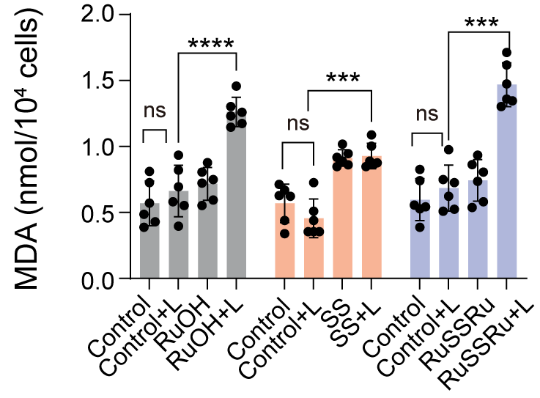


**Figure S18.** Malondialdehyde (MDA) levels of MC38 cells incubated with RuOH, SS, or RuSSRu, under dark or light irradiation (L) conditions.


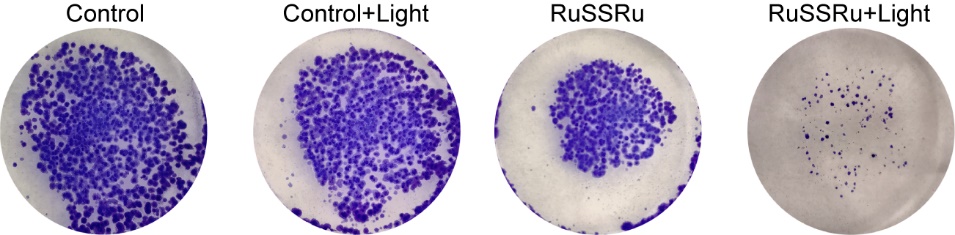


**Figure S19.** The representative images of cell colony of RuSSRu, under dark or light irradiation conditions.


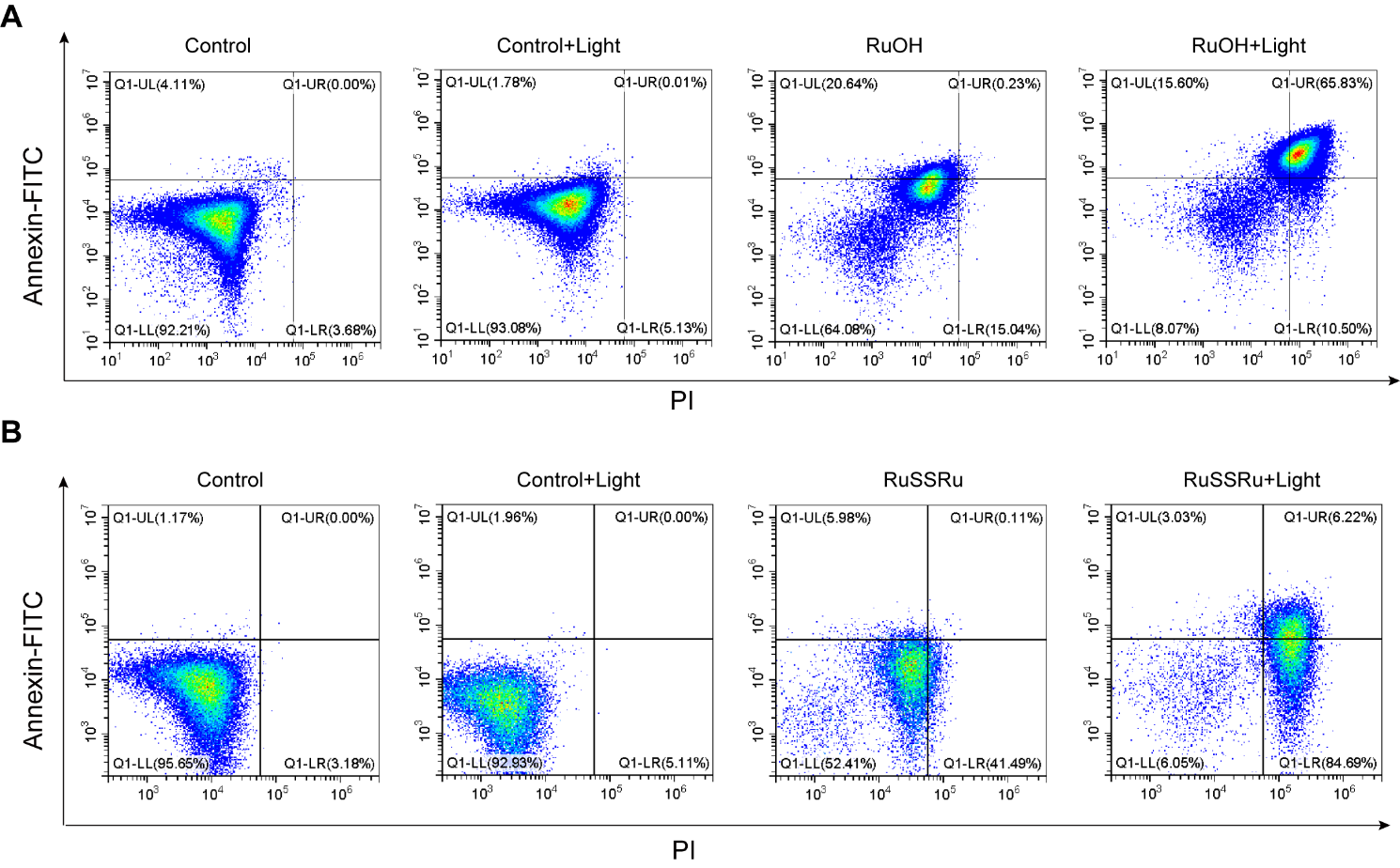


**Figure S20.** A-B) The flow cytometric analysis of apoptosis induced by RuOH and RuSSRu in different conditions.


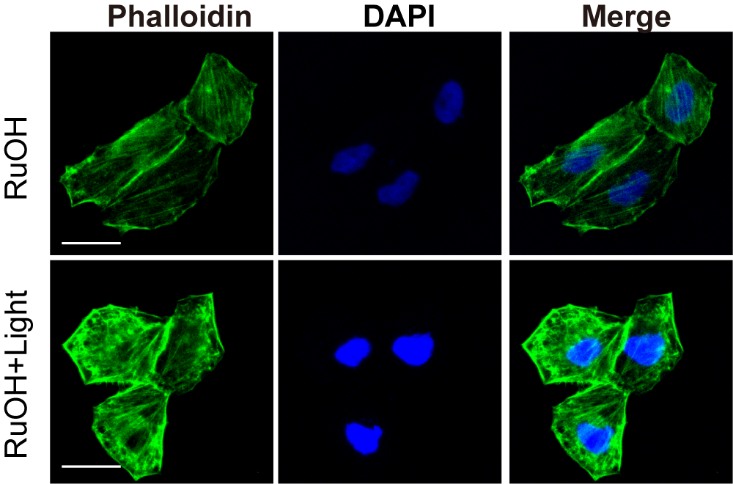


**Figure S21.** Fluorescent staining of F-actin using Alexa 488 conjugated phalloidin in MC38 cells cultured with RuOH, under dark or light irradiation conditions. Scale bar = 20 μm.


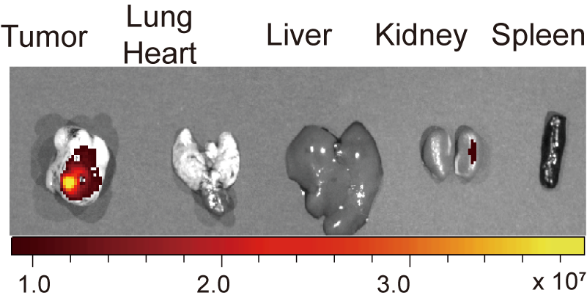


**Figure S22.** The targetability of RuSSRu in tumor and main organs (heart, lung, liver, spleen, and kidney).


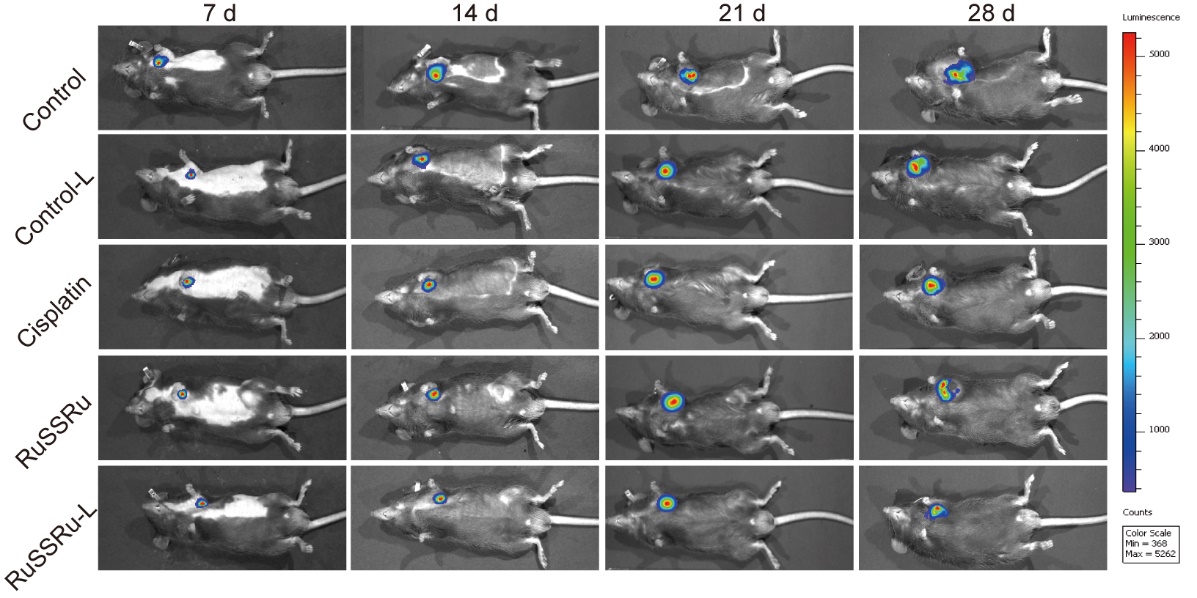


**Figure S23.** The small animal living imaging after drug treatment at 7, 14, 21, and 28 days.


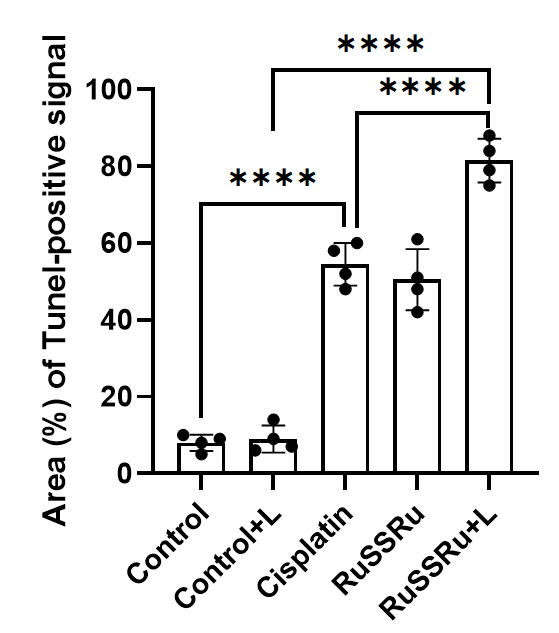


**Figure S24.** Relative quantification of TUNEL fluorescence.


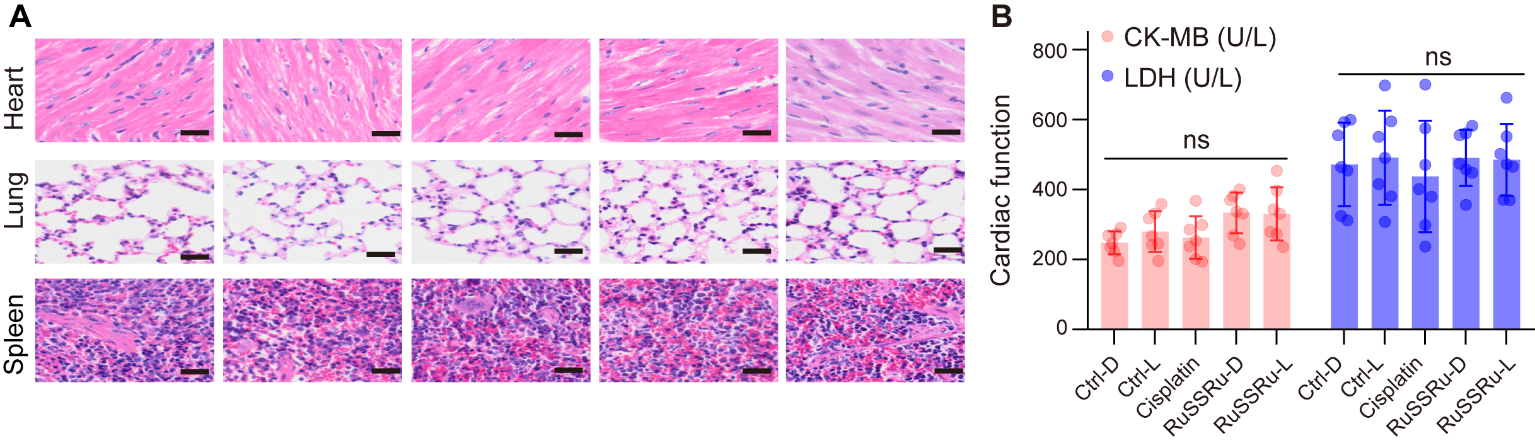


**Figure S25.** A) Representative images of H&E staining of heart, lung, and spleen. Scale bar = 40 μm. B) Cardiac functions.


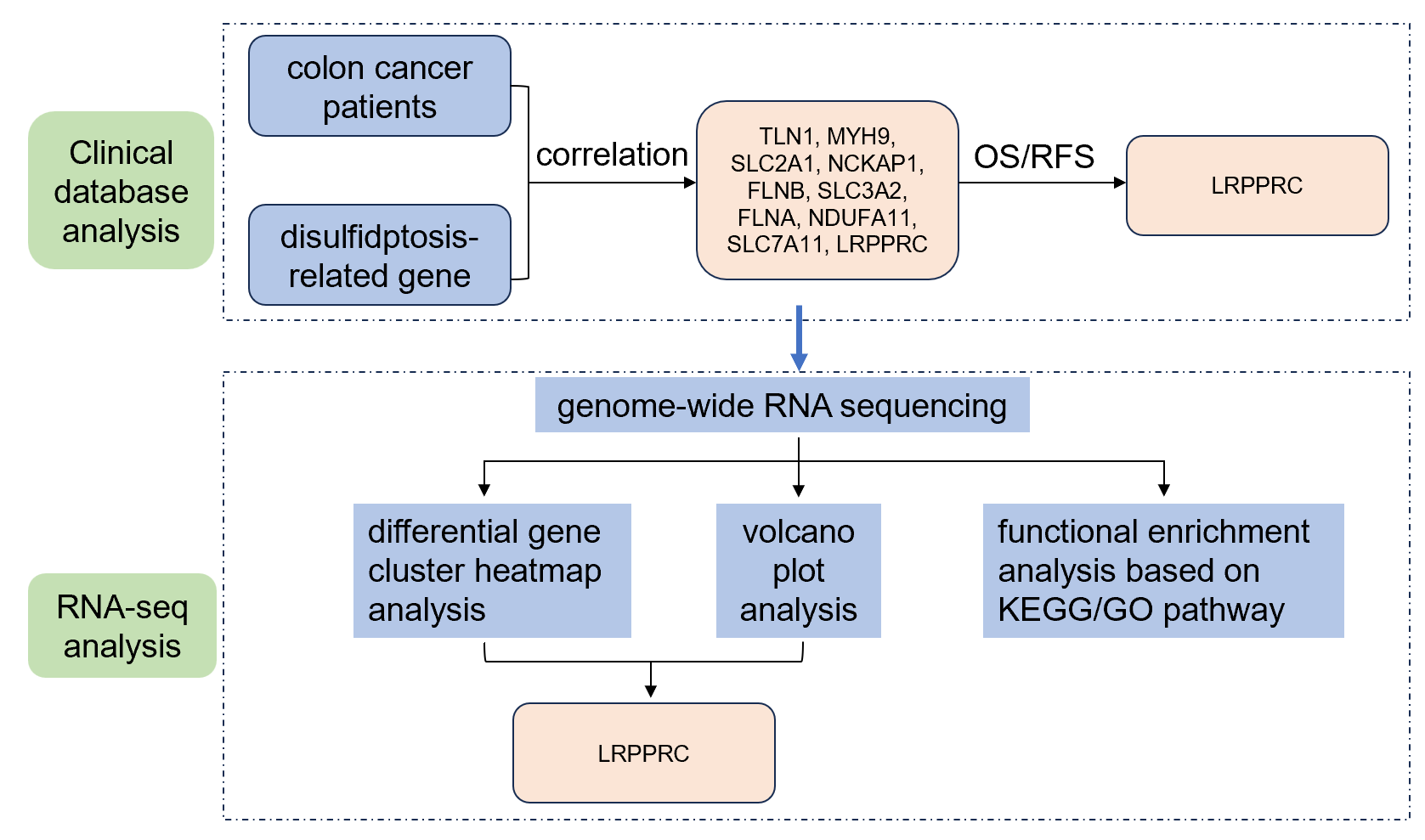


**Figure S26**. Bioinformatic analysis workflow.


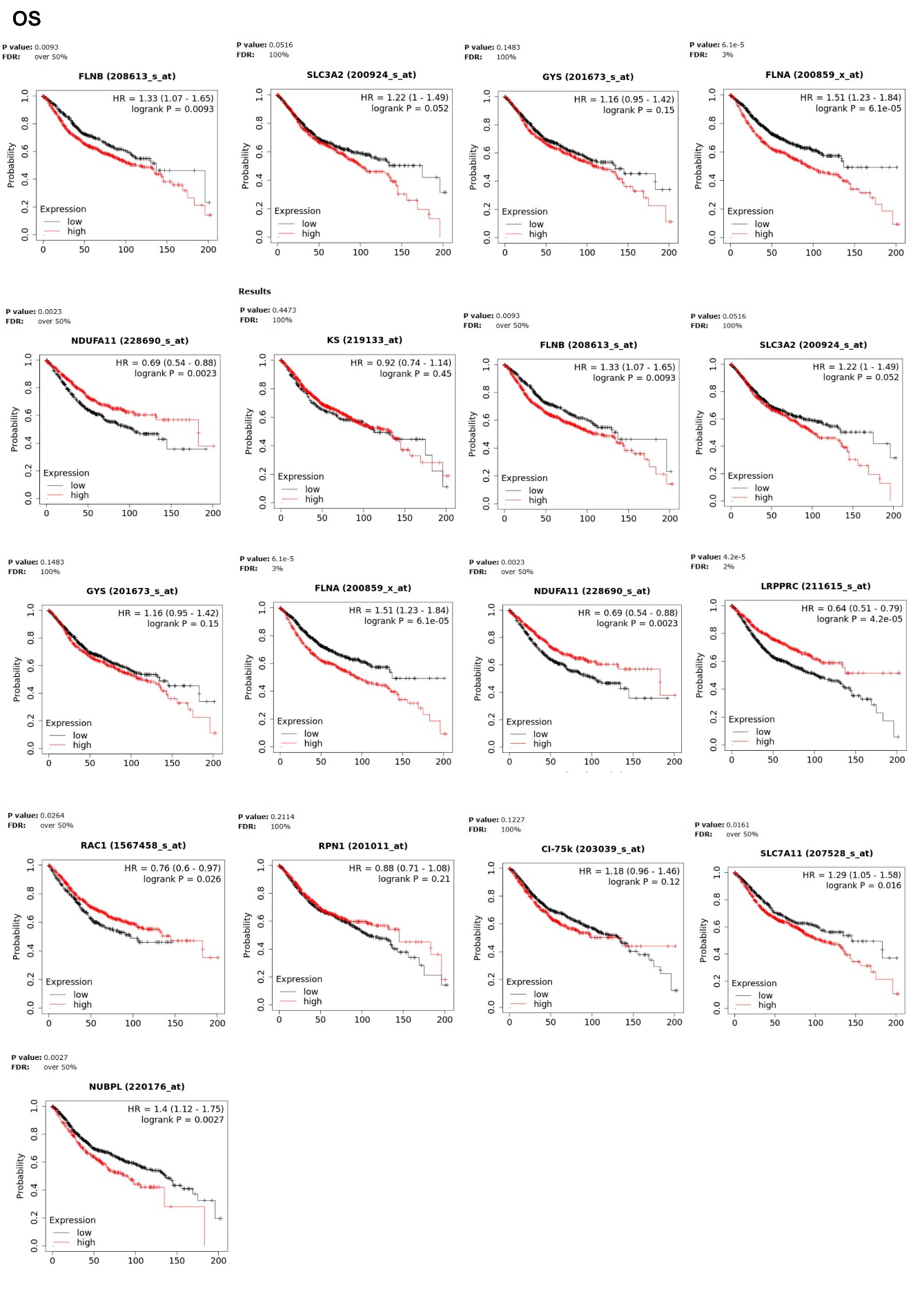


**Figure S27.** Kaplan–Meier analysis of OS based on disulfidptosis-associated genes mRNA levels using the KM-plotter colon cancer database.


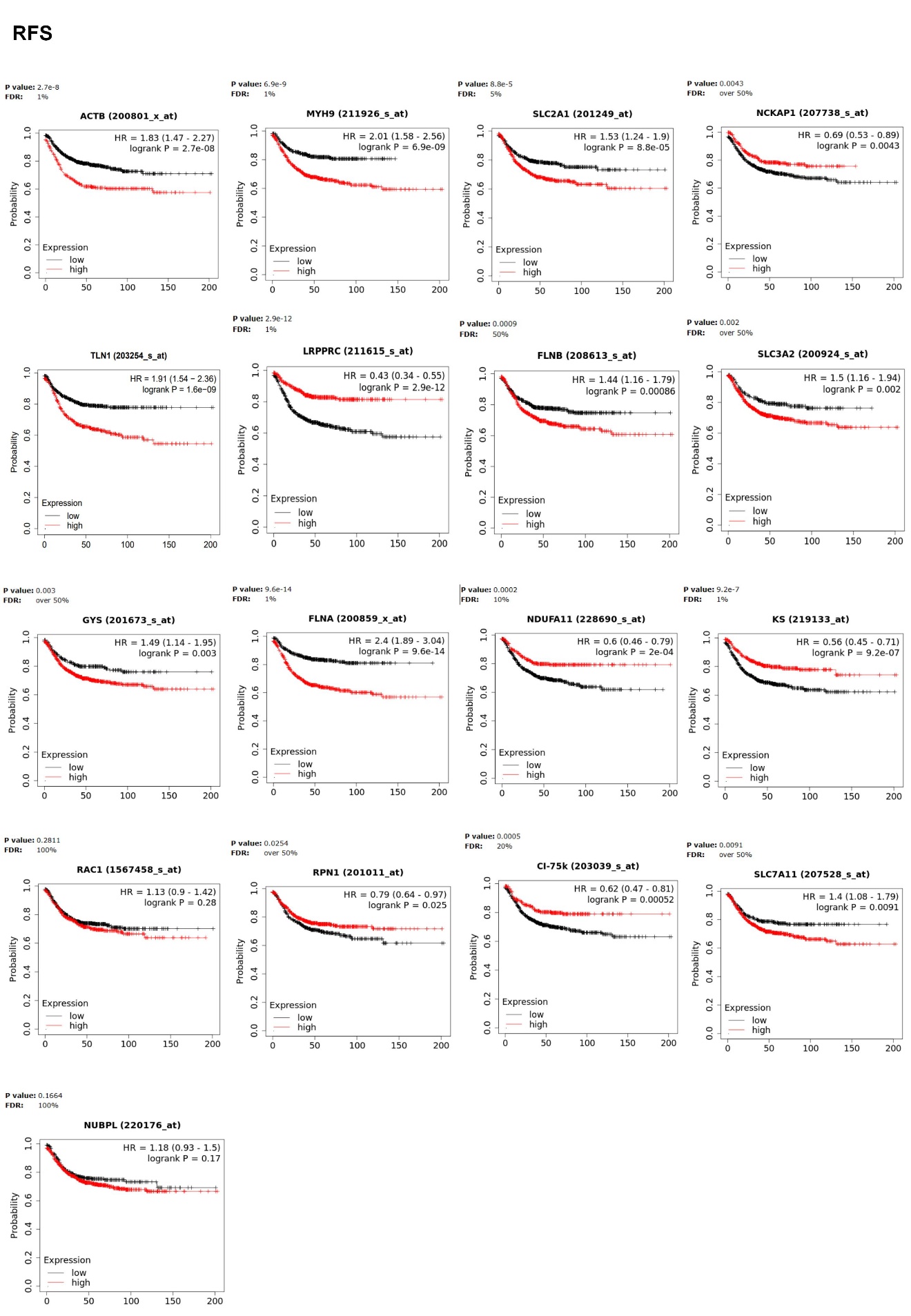


**Figure S28**. Kaplan–Meier analysis of RFS based on disulfidptosis-associated genes mRNA levels using the KM-plotter colon cancer database.


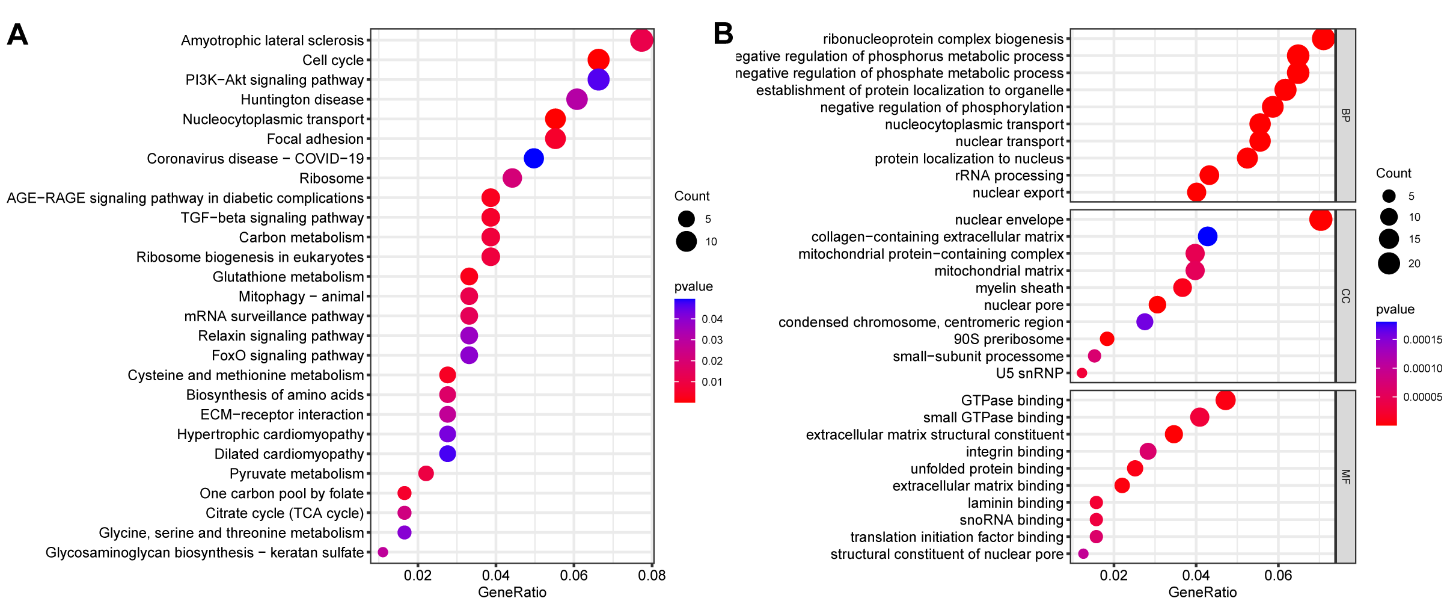


**Figure S29**. A) The enrichment analysis of Kyoto Encyclopedia of Genes and Genomes (KEGG) between different groups. B) The enrichment analysis of Gene Ontology (GO) between different groups.


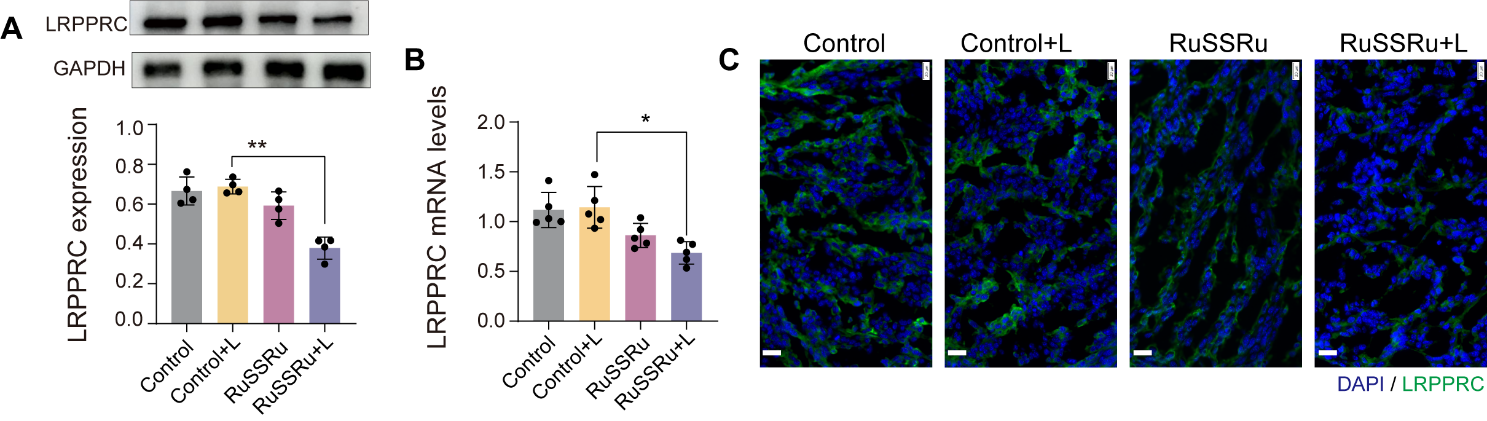


**Figure S30.** A) The expression of LRPPRC protein in tumor tissue, n = 4. B) The mRNA levels of LRPPRC in tumor tissue, n = 5. C) Representative immunofluorescent staining of LRPPRC expression in tumor. Scale bar = 20 μm.


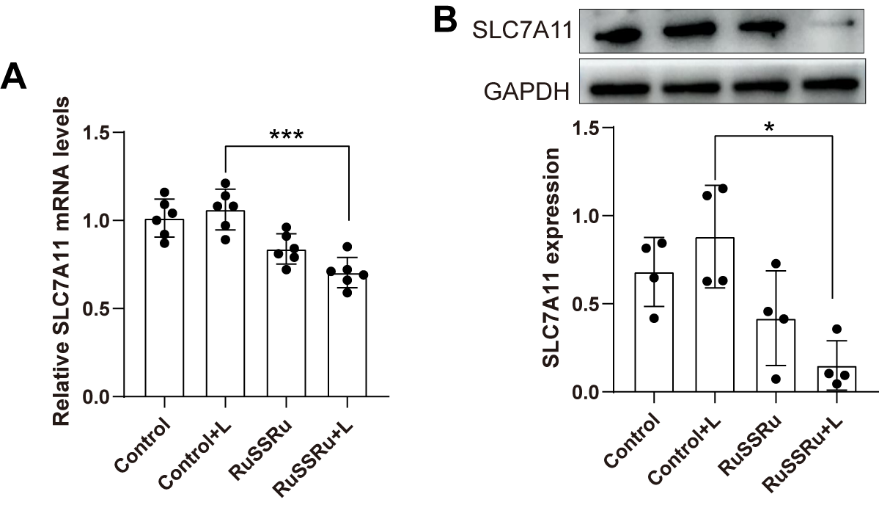


**Figure S31.** The expression of SLC7A11 under different conditions. (A), SLC7A11 mRNA levels (n = 5). (B), SLC7A11 protein expression (n = 4). **P* < 0.05, ****P* < 0.001.

**Table S1** Photophysical characterization of RuSSRu.

| Solvent | λ_abs_^[a]^ (nm) | | ε (*10^5^M^-1^cm^-1^) | λ_em_^[b]^ (nm) | Φ_f_^[c]^ (%) | Stokes shift (cm^-1^/nm) | Brightness (ε* Φ_f_) |
| --- | --- | --- | --- | --- | --- | --- | --- |
| DCM | | 454 | 0.282 | 589 | 4.65 | 5034/134.5 | 1311.3 |
| DMSO | | 456 | 0.267 | 605 | 2.09 | 5401/149 | 558 |
| Water | | 454 | 0.24 | 605 | 2.88 | 5484/150.5 | 691.2 |
| MeOH | | 452 | 0.281 | 605 | 1.23 | 5595/153 | 345.6 |

^[a]^ The absorption maximum of RuSSRu.

^[b]^ The emission maximum of RuSSRu.

^[c]^ The relative phosphorescence quantum yield by using R-6G in ethyl alcohol as a reference system.

**Reference**

1. Fan, Y. *et al.* Lifetime-engineered NIR-II nanoparticles unlock multiplexed in vivo imaging. *Nature Nanotechnology* **13** (10), 941-946 (2018).

2. Zhong, Y. *et al.* Boosting the down-shifting luminescence of rare-earth nanocrystals for biological imaging beyond 1500 nm. *Nature Communications* **8** (1), 737, (2017).

3. Brouwer, A. M. Standards for photoluminescence quantum yield measurements in solution (IUPAC Technical Report). *Pure and Applied Chemistry* **83** (12), 2213-2228 (2011).

4. Makarov, N. S., Drobizhev, M. & Rebane, A. Two-photon absorption standards in the 550-1600 nm excitation wavelength range. *Optics Express* **16** (6), 4029-4047 (2008).

5. Zhuang, J. *et al.* Thymoquinone as an electron transfer mediator to convert Type II photosensitizers to Type I photosensitizers. *Nature Communications* **15** (1), 4943, (2024).

**Original data of Western blot:**

**1. Uncropped/full-size gel/blot for Figure 4 (Bax, n = 3).**


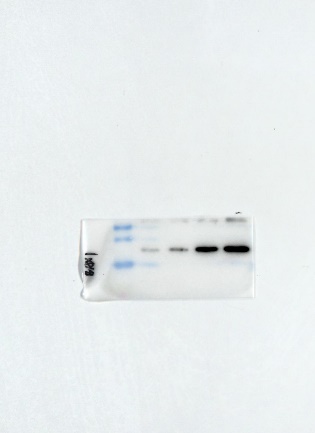

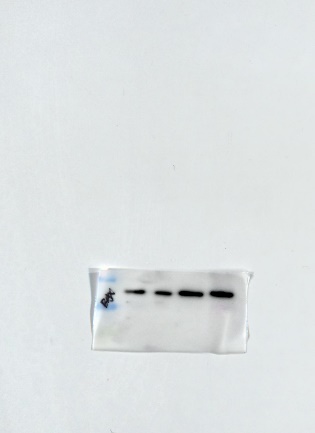

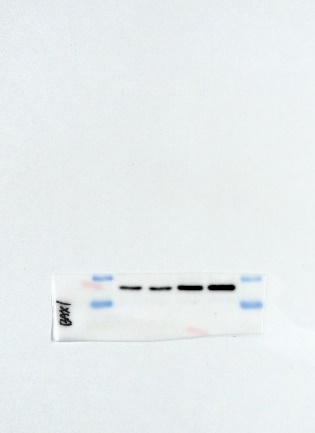


Protein: Bax; Molecular weight: ~21 kDa

The band from left to right was: Control, Control+Light, RuSSRu, RuSSRu+Light.

**2. Uncropped/full-size gel/blot for Figure 4 (BCL-2, n = 6).**


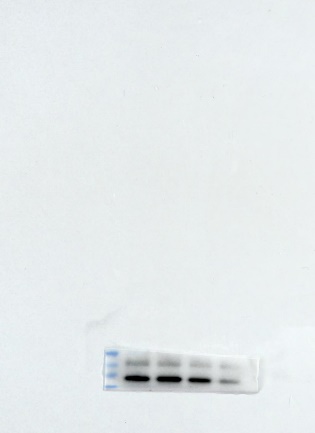

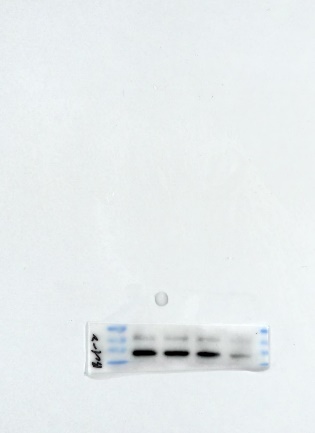

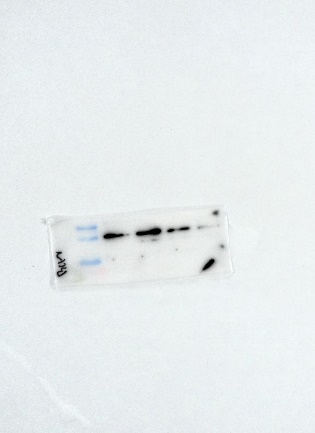

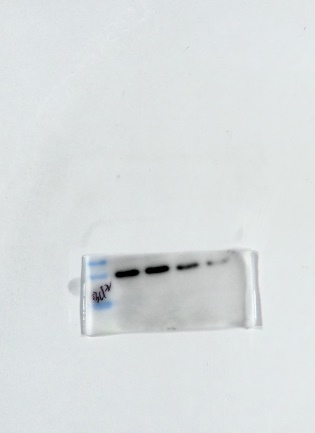


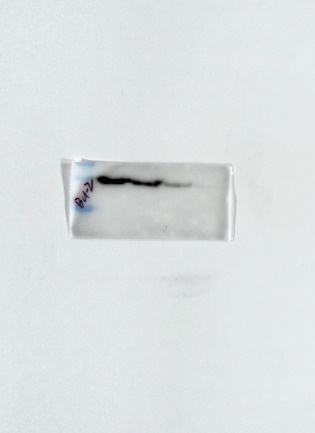

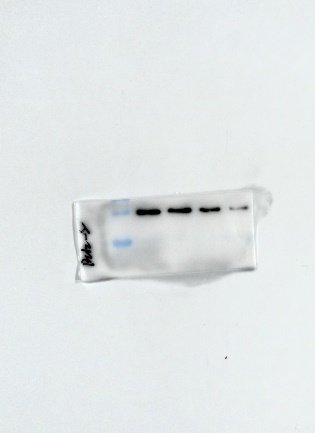


Protein: BCL-2; Molecular weight: ~26 kDa

The band from left to right was: Control, Control+Light, RuSSRu, RuSSRu+Light.

**3. Uncropped/full-size gel/blot for Figure 4 (Cleaved-caspase 3, n = 6).**


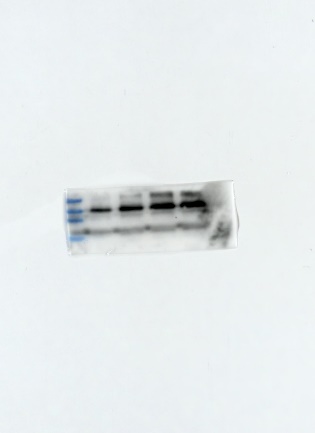

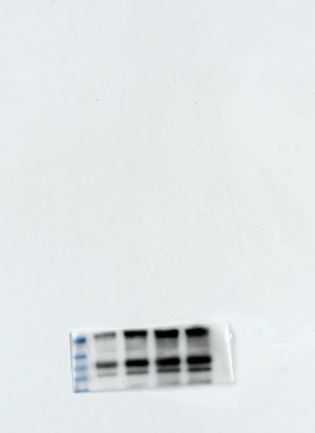

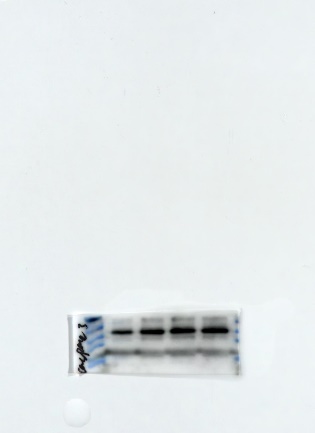

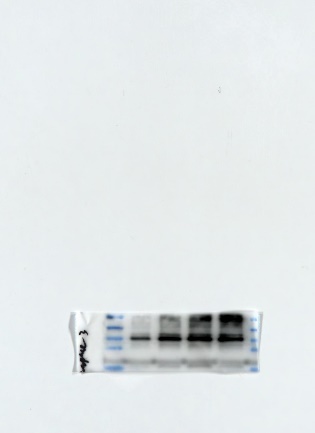

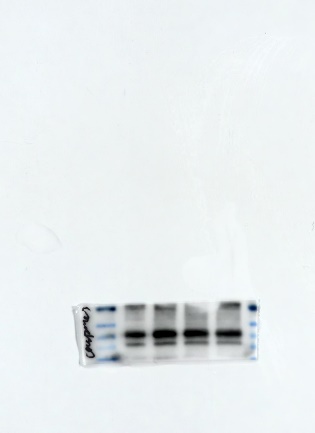

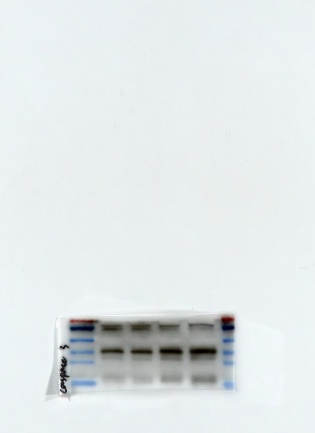


Protein: Cleaved-caspase 3; Molecular weight: ~31 kDa

The band from left to right was: Control, Control+Light, RuSSRu, RuSSRu+Light.

**4. Uncropped/full-size gel/blot for Figure 7 (LRPPRC for cells, n = 4).**


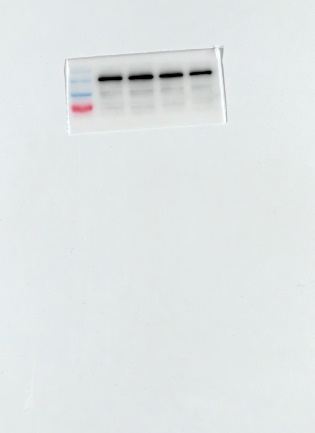

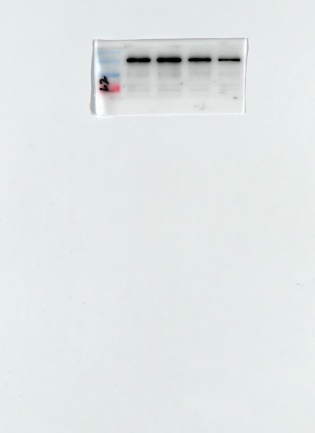

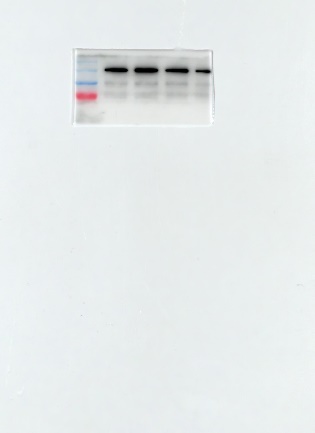

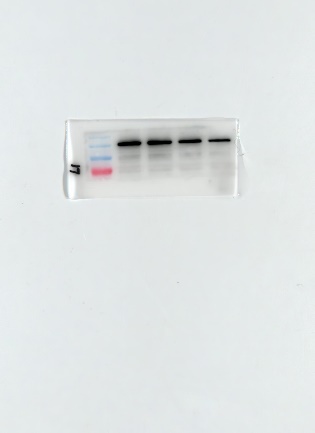


Protein: LRPPRC; Molecular weight: ~130 kDa

The band from left to right was: Control, Control+Light, RuSSRu, RuSSRu+Light.

**5. Uncropped/full-size gel/blot for Figure S11 (Nuclear TFEB, n = 7).**


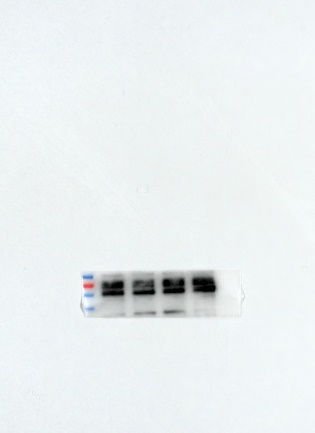

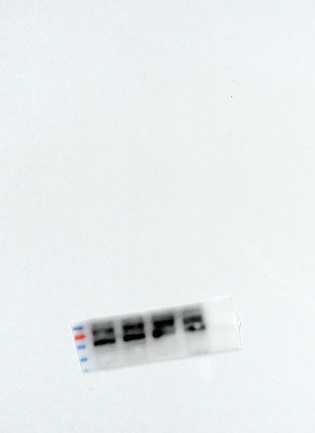

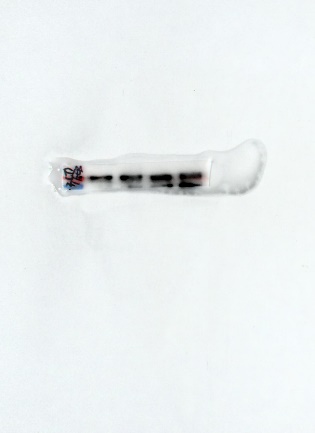

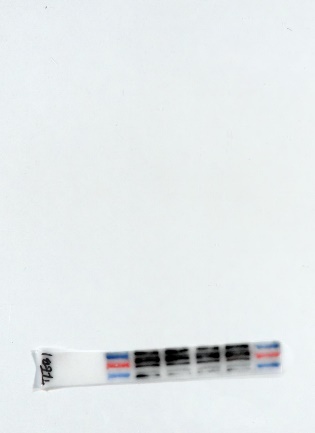


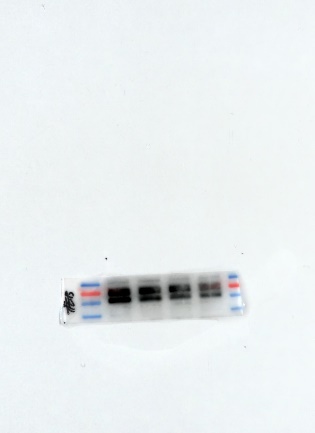

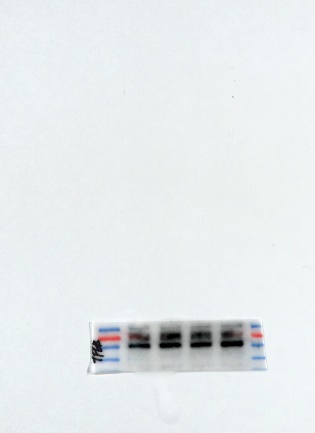

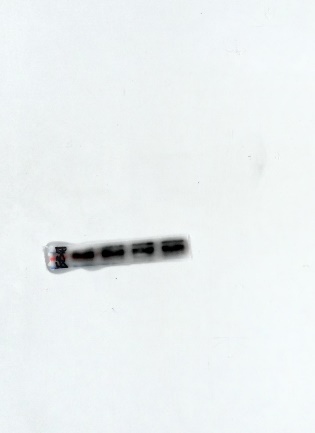


Protein: TFEB; Molecular weight: ~65 kDa

The band from left to right was: Control, Control+Light, RuSSRu, RuSSRu+Light.

**6. Uncropped/full-size gel/blot for Figure S11 (Total TFEB, n = 8).**


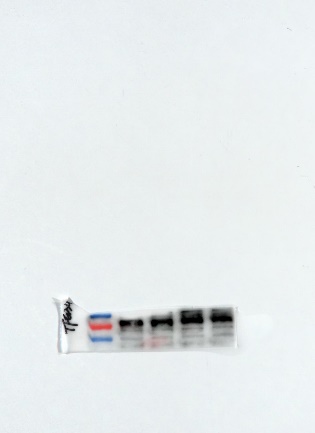

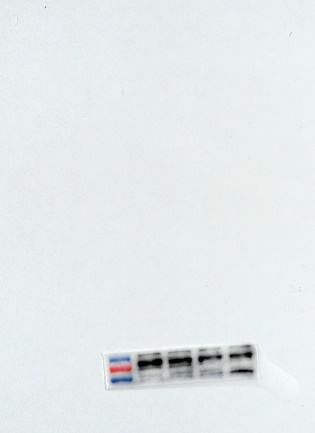

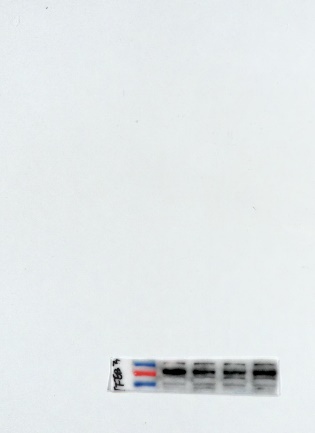

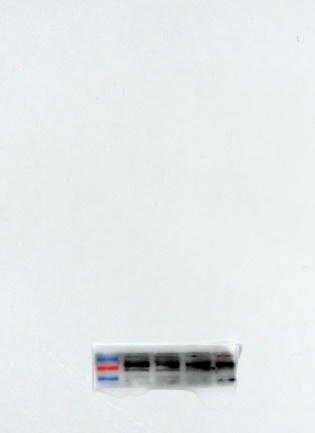


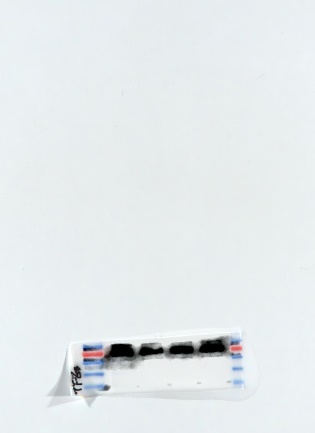

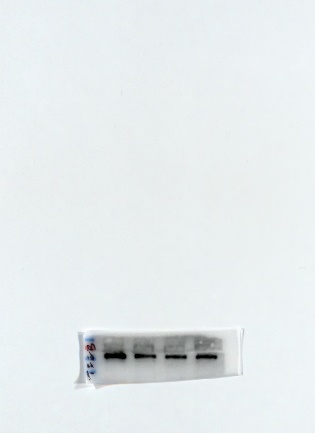

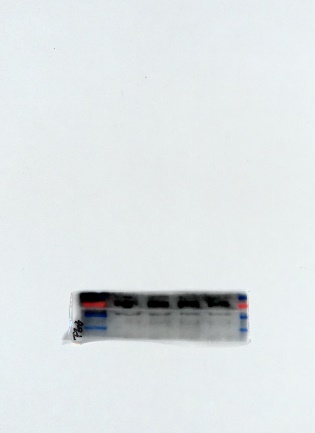

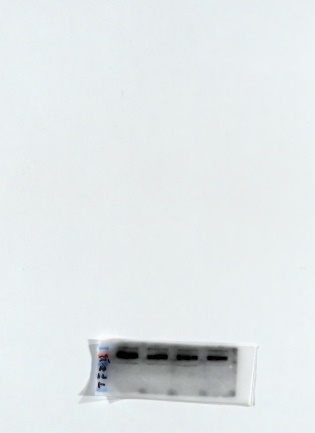


Protein: TFEB; Molecular weight: ~65 kDa

The band from left to right was: Control, Control+Light, RuSSRu, RuSSRu+Light.

**7. Uncropped/full-size gel/blot for Figure S11 (Tubulin, n = 5).**


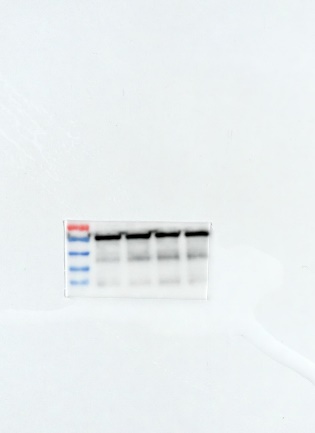

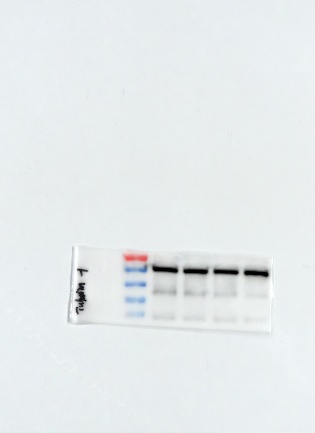

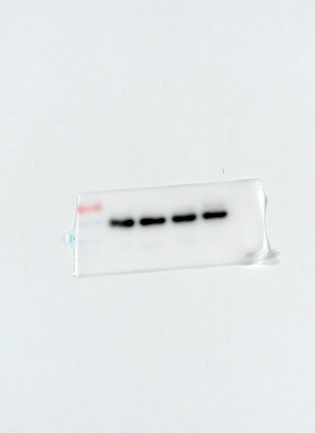

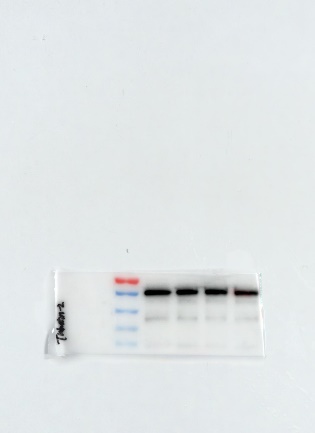

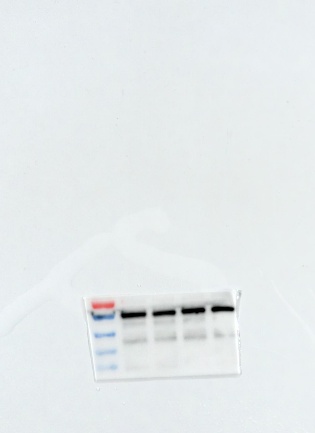


Protein: Tubulin; Molecular weight: ~50 kDa

The band from left to right was: Control, Control+Light, RuSSRu, RuSSRu+Light.

**8. Uncropped/full-size gel/blot for Figure S30 (LRPPRC for tumor tissue, n = 4).**


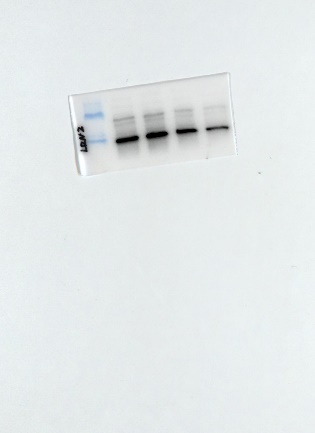

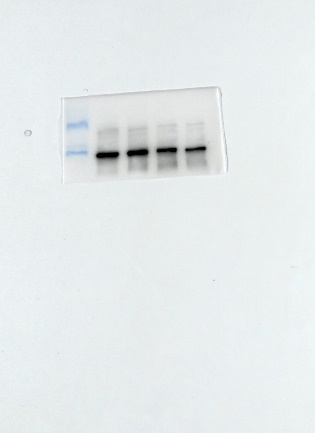

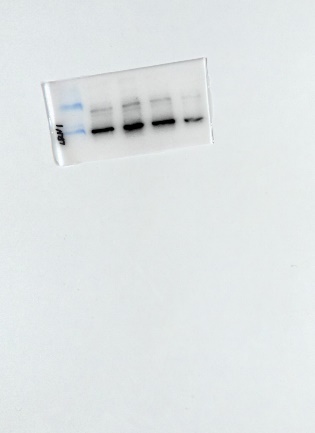

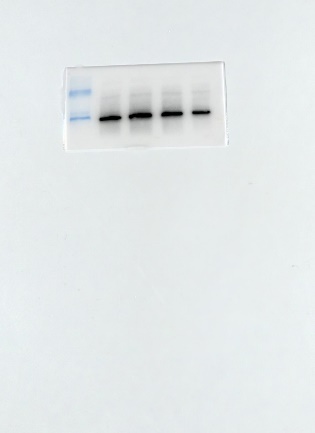


Protein: LRPPRC; Molecular weight: ~130 kDa

The band from left to right was: Control, Control+Light, RuSSRu, RuSSRu+Light.

**9. Uncropped/full-size gel/blot for Figure S31 (SLC7A11, n = 4).**


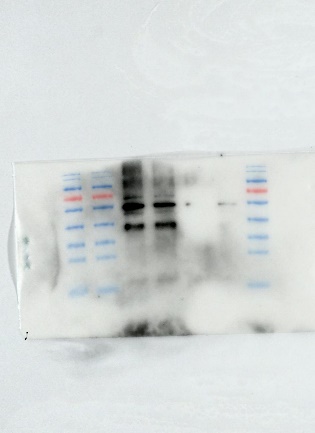

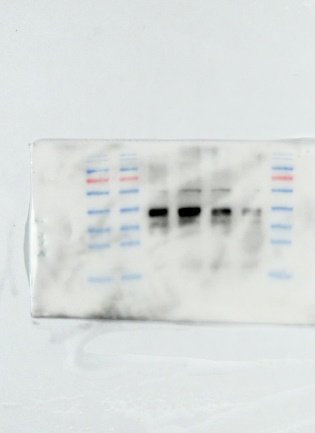

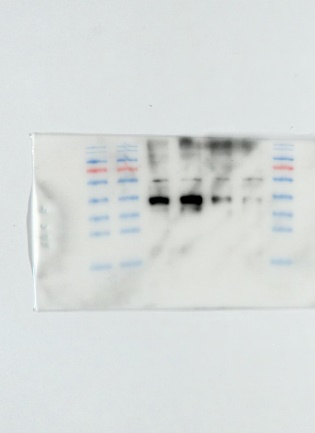

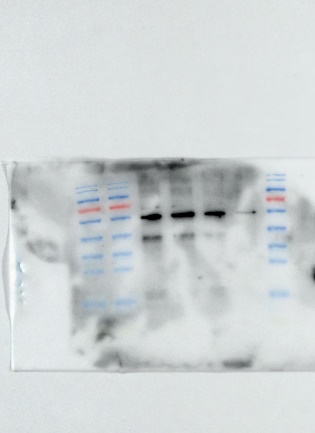


Protein: SLC7A11; Molecular weight: ~35 kDa

The band from left to right was: Control, Control+Light, RuSSRu, RuSSRu+Light.

**10. GAPDH for cells (n = 8).**


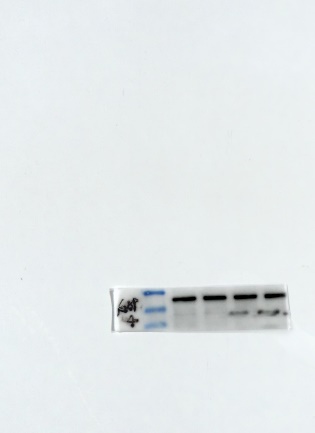

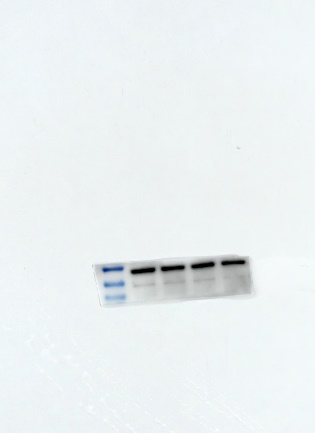

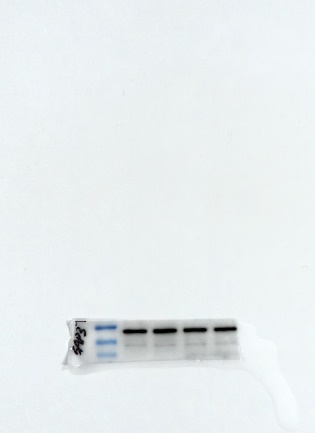

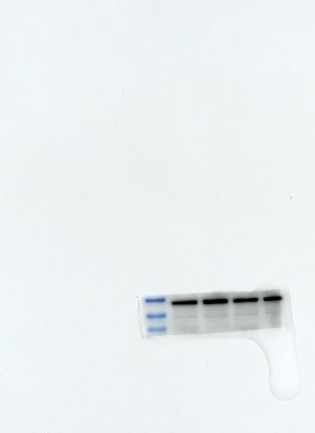


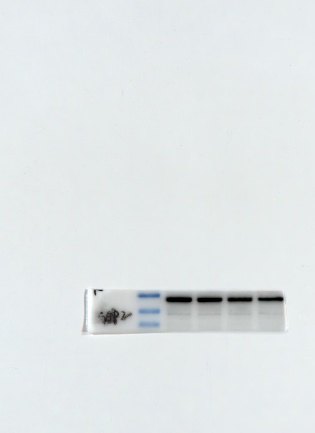

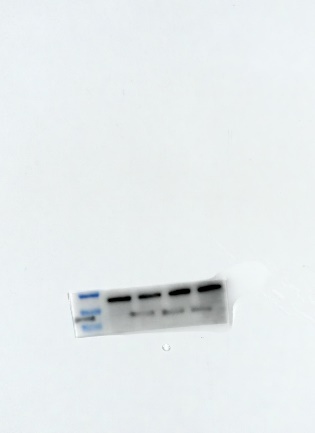

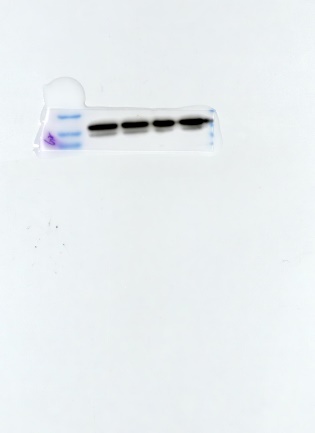

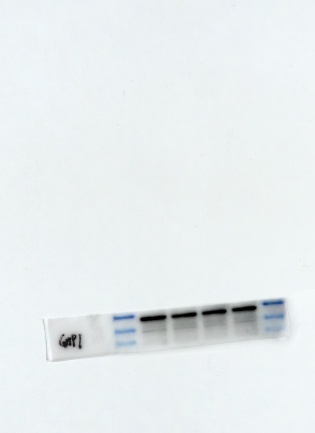


Protein: GAPDH; Molecular weight: ~35 kDa

The band from left to right was: Control, Control+Light, RuSSRu, RuSSRu+Light.

**11. GAPDH for tumor tissue (n = 4).**


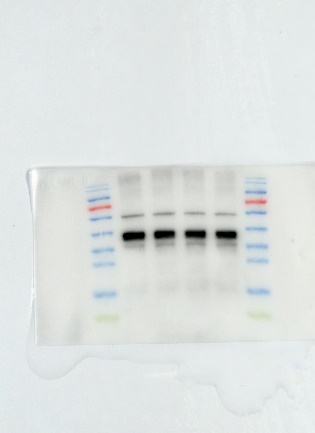

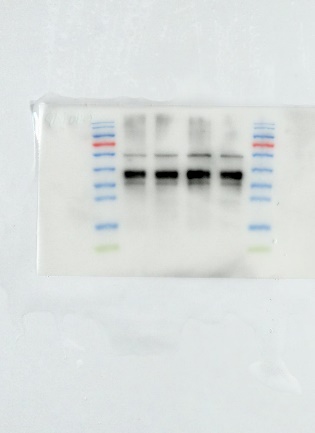

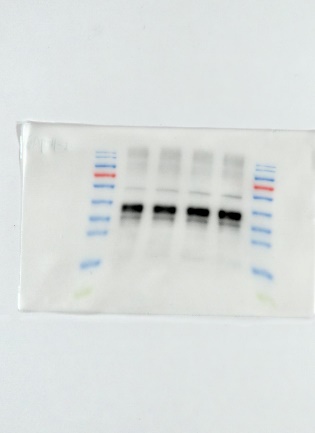

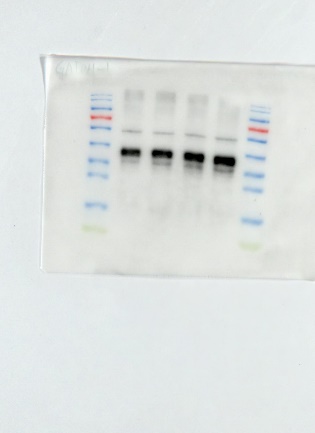


Protein: GAPDH; Molecular weight: ~36 kDa

The band from left to right was: Control, Control+Light, RuSSRu, RuSSRu+Light.
